# Evaluating the cost-effectiveness of COVID-19 mRNA primary-series vaccination in Qatar: An integrated epidemiological and economic analysis

**DOI:** 10.1371/journal.pone.0331654

**Published:** 2025-09-26

**Authors:** Hiam Chemaitelly, Houssein H. Ayoub, Peter Coyle, Patrick Tang, Mohammad R. Hasan, Hadi M. Yassine, Asmaa A. Al Thani, Zaina Al-Kanaani, Einas Al-Kuwari, Andrew Jeremijenko, Anvar Hassan Kaleeckal, Ali Nizar Latif, Riyazuddin Mohammad Shaik, Hanan F. Abdul-Rahim, Gheyath K. Nasrallah, Mohamed Ghaith Al-Kuwari, Adeel A. Butt, Hamad Eid Al-Romaihi, Mohamed H. Al-Thani, Abdullatif Al-Khal, Roberto Bertollini, Laith J. Abu-Raddad

**Affiliations:** 1 Infectious Disease Epidemiology Group, Weill Cornell Medicine-Qatar, Cornell University, Doha, Qatar; 2 Department of Health Policy, London School of Economics, London, United Kingdom; 3 Department of Population Health Sciences, Weill Cornell Medicine, Cornell University, New York, New York, United States of America; 4 Mathematics Program, Department of Mathematics, Statistics, and Physics, College of Arts and Sciences, Qatar University, Doha, Qatar; 5 Hamad Medical Corporation, Doha, Qatar; 6 Department of Biomedical Science, College of Health Sciences, QU Health, Qatar University, Doha, Qatar; 7 Wellcome-Wolfson Institute for Experimental Medicine, Queens University, Belfast, United Kingdom; 8 Department of Pathology and Laboratory Medicine, University of British Columbia, Vancouver, Canada; 9 Department of Pathology and Molecular Medicine, McMaster University, Hamilton, Canada; 10 Biomedical Research Center, QU Health, Qatar University, Doha, Qatar; 11 Department of Public Health, College of Health Sciences, QU Health, Qatar University, Doha, Qatar; 12 Primary Health Care Corporation, Doha, Qatar; 13 College of Medicine, Qatar University, Doha, Qatar; 14 Department of Medicine, Weill Cornell Medicine, Cornell University, New York, New York, United States of America; 15 Ministry of Public Health, Doha, Qatar; 16 College of Health and Life Sciences, Hamad bin Khalifa University, Doha, Qatar; UniCamillus: Saint Camillus International University of Health and Medical Sciences, ITALY

## Abstract

Qatar implemented a mass primary-series vaccination campaign to mitigate the impact of the coronavirus disease 2019 (COVID-19) pandemic. This study aimed to retrospectively evaluate the cost-effectiveness of this program both before and after onset of the omicron wave. An economic evaluation was conducted from the public healthcare system perspective between January 5, 2021, and September 18, 2023. Cost-effectiveness was determined using an epidemiological retrospective cohort study and health economic modeling that compared the cohort of individuals who received two vaccine doses with the unvaccinated cohort with respect to incidence of infection, incidence of severe COVID-19 forms, quality-adjusted life years (QALYs), and medical costs. During the pre-omicron phase, primary-series vaccination incurred an additional cost of $104,422,358, led to a gain of 724.7 QALYs, and savings of $54,790,858 in direct medical costs. The incremental cost-effectiveness ratio (ICER) was $68,485 per QALY gained. The number needed to vaccinate was 35.4 individuals (95% CI: 24.4–49.9) to prevent one infection and 718.0 individuals (95% CI: 469.4–984.0) to prevent one severe COVID-19 outcome. The cost per infection averted was $3,180 (95% CI: $2,189-$4,484) and per severe COVID-19 outcome averted was $64,468 (95% CI: $42,146-$88,354). Vaccination of individuals ≥50 years of age, those more clinically vulnerable to severe COVID-19, and those with multiple coexisting conditions was substantially more cost-effective. Cost-effectiveness of primary-series vaccination was substantially reduced during the omicron phase, but vaccination remained cost-effective. Sensitivity analyses confirmed the findings. Primary-series vaccination was cost-effective with an ICER below the 1 GDP per capita threshold during the pre-omicron phase and within the 1–3 GDP per capita thresholds during the omicron phase. Targeted vaccination strategies for those most vulnerable to COVID-19 were the most cost-effective and remained essential, even in situations of moderate vaccine effectiveness or reduced infection severity.

## Introduction

The coronavirus disease 2019 (COVID-19) pandemic, caused by severe acute respiratory syndrome coronavirus 2 (SARS-CoV-2), stands as one of the most challenging health and economic crises in recent history [1,2]. The crisis exerted unprecedented pressure on healthcare systems and resulted in extensive losses to both local and global economies [1–3]. As of March 3, 2024, the World Health Organization (WHO) estimated documented infections to exceed 774 million, with over 7 million COVID-19-related deaths [4]. Simultaneously, the International Monetary Fund estimated COVID-19 cumulative economic losses at US$13.8 trillion in 2024 [5].

The introduction of vaccination with COVID-19 mRNA vaccines, namely BNT162b2 (Pfizer-BioNTech) [6] and mRNA-1273 (Moderna) [7], marked a turning point in the pandemic. These vaccines played a pivotal role in reducing COVID-19 hospitalizations and deaths [8]. A mathematical modelling analysis suggested that, in the first year of vaccination, vaccinations averted around 20 million COVID-19-related deaths globally [9]. In an analysis focused on the United States, vaccination was estimated to have prevented close to 27 million infections, 1.6 million hospitalizations, and 235,000 COVID-19 related deaths in the first year of vaccination [10]. However, despite the rapid waning in vaccine protection against infection [11–15], and the modest and quickly waning effectiveness against the omicron variant [16], as well as its subvariants [17–19], the vaccines maintained robust protection against severe COVID-19 outcomes over time [11,14,17–19].

While the impact of vaccination on averting infection acquisition and severe forms of COVID-19 has been extensively investigated in published studies [20], economic evaluation studies remain relatively scarce. Global systematic reviews identified fewer than 30 published economic evaluation studies for COVID-19 vaccination [21,22], These studies, typically spanning one year or less, consistently demonstrated that vaccination programs using mRNA vaccines were cost-effective compared to no vaccination [21,22]. Prioritizing individuals at higher risk of infection and severe COVID-19 increased program cost-effectiveness and was in some instances cost-saving [21,22]. However, within these studies, only two originated from the Middle East and North Africa (MENA) region—one from Iran [23] and another from the Sindh province in Pakistan [24], highlighting a significant research gap in a region that accounts for 10% of the world’s population [25].

Qatar has a diverse and predominantly young population of 2.8 million, with only 9% being 50 years or older [26]. The country has undergone several phases of the SARS-CoV-2 pandemic, starting with the ancestral virus wave [26], followed by consecutive alpha [27] and beta [28] waves, a low-incidence phase dominated by delta [29], a massive omicron BA.1 and BA.2 wave [30], and subsequently a series of waves dominated by different omicron subvariants [31–33] (S1 Fig). Although numerous vaccine effectiveness studies have been conducted to inform Qatar’s COVID-19 national response [11,14,18,29,34–42], no vaccine cost-effectiveness studies have been conducted to date.

The objective of this study was to fill this gap by conducting an economic evaluation of the primary-series COVID-19 mRNA vaccination in Qatar’s predominantly young population. The evaluation spans both the pre- and post-omicron periods, assessing the retrospective cost-effectiveness of the vaccination campaign in the context of evolving epidemiological conditions, viral evolution, and changes in vaccine effectiveness. The ultimate goal is to provide insights into the experience of COVID-19 vaccination, enabling efficient resource allocation in vaccination programs, and informing future preparedness plans.

## Methods

### Study population, data sources, and vaccination

This study investigated the cost-effectiveness of COVID-19 mRNA primary-series vaccination in Qatar’s population from the perspective of the public healthcare system, the exclusive provider of COVID-19 vaccination in the country. The evaluation was conducted between January 5, 2021, date of first completed primary-series vaccination in Qatar, and September 18, 2023, the study end date. Cost-effectiveness was analyzed separately for the pre-omicron and omicron phases of the pandemic, considering the substantial reduction in vaccine effectiveness after the introduction of the omicron [17,18].

The study analyzed the national, federated databases for COVID-19 laboratory testing, vaccination, hospitalization, and death, retrieved from the integrated, nationwide, digital-health information platform (Section S1 in ,S1 file). These databases contain SARS-CoV-2-related data with no missing information since the pandemic’s onset, including all polymerase chain reaction (PCR) tests irrespective of location or facility. The databases also include all medically supervised rapid antigen tests conducted from January 5, 2022, when rapid antigen testing was introduced into the national strategy to alleviate pressure on PCR testing during the peak of the first omicron wave, which was larger than all preceding waves (Section S1). Qatar maintained an extensive testing approach up to October 31, 2022, with nearly 5% of the population tested every week, primarily for routine reasons, such as screening or travel [11,17]. Starting from November 1, 2022, testing was reduced to below 1% of the population being tested every week up to the end of the study. Most infections were identified through routine testing rather than symptom-based testing (Sections S1 and S2) [11,17].

Mass COVID-19 vaccination started in Qatar on December 21, 2020 using BNT162b2, followed by mRNA-1273 three months later [37]. Vaccination was delivered free of charge regardless of citizenship status exclusively through the public healthcare system [34]. Vaccination rollout prioritized frontline healthcare workers, individuals with severe or multiple chronic conditions, and individuals aged 50 years or older [11]. Vaccination of adolescents aged 12–17 years started in February of 2021 using the same BNT162b2 vaccine as that for adults (30 μg antigen dose), whereas vaccination of children aged 5–11 years was initiated in February 2022 using the 10 μg BNT162b2 vaccine [40,41]. Immunizations were implemented, throughout the pandemic, adhering strictly to the United States Food and Drug Administration approved protocol [6,7].

Demographic information pertaining to sex, age, and nationality were extracted as recorded in the national health registry. Qatar has distinct demographics with 89% of its population being expatriates from over 150 countries [26]. Further details on Qatar’s population and national COVID-19 databases have been previously published [11,17,26,39,42–44].

### Study design

Two retrospective cohort studies were conducted to assess the cost-effectiveness of primary-series vaccination in averting SARS-CoV-2 infection and associated severe, critical, or fatal COVID-19 before and after onset of omicron phase in Qatar. Cost-effectiveness was determined by comparing the cohort of individuals who received the primary-series (designated the two-dose cohort) to the cohort of unvaccinated persons (designated the unvaccinated/control cohort) with respect to incidence of infection, progression to severe forms of COVID-19, quality-adjusted life years (QALYs), and medical costs.

Incidence of infection was defined as any PCR-positive or rapid-antigen-positive test after the start of follow-up, regardless of symptoms. Infection severity classification followed the WHO guidelines for COVID-19 case severity (acute-care hospitalizations) [45], criticality (intensive-care-unit hospitalizations) [45,46], and fatality [47] (Section S3). Individuals whose infection progressed to severe, critical, or fatal COVID-19 were classified based on their worst assessment outcome, starting with COVID-19 death [47], followed by critical disease [45], and then severe disease [45] (Section S3). Incidence of severe COVID-19 outcomes was recorded on the date of the SARS-CoV-2-positive test confirming the infection.

All individuals who received two mRNA vaccine doses were eligible for inclusion in the study provided they had no documented SARS-CoV-2 infection in the 90 days preceding their second vaccine dose. The 90-day threshold was set to avoid misclassification of a previous SARS-CoV-2 infection as an incident infection if a shorter timeframe was used [17,48,49]. Individuals who received their second dose in a specific calendar week in the two-dose cohort were matched exactly one-to-one to individuals in the control cohort who had a record for a SARS-CoV-2-negative test in that same calendar week. This strategy guaranteed that matched pairs were present in Qatar during the same time period. An iterative selection algorithm was implemented to ensure that controls were unvaccinated at the start of follow-up and had no documented SARS-CoV-2 infection in the 90 days prior to the start of follow-up (Section S4). Each pair was followed from the date of the second dose for the individual in the two-dose cohort.

For exchangeability [42,50], both individuals of each matched pair were censored at the earliest occurrence of a person receiving a new vaccine dose. Consequently, individuals were followed up until the first of any of the following events: a documented SARS-CoV-2 infection (regardless of symptoms), or third-dose vaccination for persons in the two-dose cohort (with matched-pair censoring), or first-dose vaccination for persons in the unvaccinated cohort (with matched-pair censoring), or death, or end of study.

Considering a durability for vaccine protection against infection of about one year during the pre-omicron phase [11–15,51] and 6 months during the omicron phase [17–19] (which began on December 19, 2021 [52]), the end of study was set on December 18, 2021 for the pre-omicron phase, and either 6 months after the second dose or September 18, 2023 (end of study), whichever occurred first, for the omicron phase.

### Oversight

The institutional review boards at Hamad Medical Corporation and Weill Cornell Medicine–Qatar approved this retrospective study with a waiver of informed consent. Data were provided to the researchers through a restricted-access agreement that prevents sharing the dataset with a third party or publicly and preserves the confidentiality of identifiable patient data. The study was reported according to the Strengthening the Reporting of Observational Studies in Epidemiology (STROBE; S1 Table in S1 File) and the Consolidated Health Economic Evaluation Reporting Standards (CHEERS; S2 Table in S1 File) guidelines.

### Statistical analysis

#### Descriptive statistics and incidence.

Eligible and matched cohorts were described using frequency distributions and measures of central tendency and were compared using standardized mean differences (SMDs). Cumulative incidence of infection (or of severe, critical, or fatal COVID-19), defined as the proportion of persons at risk whose primary endpoint during follow-up was an infection (or severe COVID-19 outcomes), was estimated using the Kaplan-Meier estimator method.

Incidence rate of infection (or severe COVID-19 outcomes) in each cohort, defined as number of identified infections (or severe COVID-19 outcomes) divided by number of person-weeks contributed by all individuals in the cohort, was estimated, with the corresponding 95% confidence interval (CI), using a Poisson log-likelihood regression model with Stata 18.0 *stptime* command.

#### Estimation of the number needed to vaccinate.

Cox regression models were applied, using Stata 18.0 *stcox* command, to calculate adjusted hazard ratios (AHR) that compare incidence of infection (or severe COVID-19 outcome) between the cohorts, adjusted for sex, age, nationality, and number of coexisting medical conditions (groups shown in Tables 1,2). Interactions were not considered. Regression coefficients were then used to determine the baseline survival probability for each individual and estimate the average survival probability in the study population assuming that no one had received vaccination, ${\overset{―}{\text{S}}}_{\text{unvaccinated}}(\text{t}$[53]. Regression coefficients were also used to determine the survival probability for each individual and estimate the average survival probability in the study population assuming that all individuals had received primary-series vaccination, ${{\overset{―}{\text{S}}}_{\text{unvaccinated}}(\text{t})}^{AHR}$ [53].

**Table 1:** Baseline characteristics of eligible and matched cohorts.

Characteristics	Pre-omicron phase						Omicron phase					
	Full eligible cohorts			Matched cohorts*			Full eligible cohorts			Matched cohorts*		
	Two-dose	Unvaccinated	SMD^†^	Two-dose	Unvaccinated	SMD^†^	Two-dose	Unvaccinated	SMD^†^	Two-dose	Unvaccinated	SMD^†^
	N = 1,860,778	N = 2,642,137		N = 1,162,962	N = 1,162,962		N = 79,412	N = 2,578,075		N = 79,409	N = 79,409	
Median age (IQR)—years	35.0 (29.0-43.0)	33.0 (25.0-41.0)	0.32^‡^	35.0 (28.0-44.0)	30.0 (21.0-38.0)	0.57^‡^	18.0 (9.0-33.0)	33.0 (24.0-41.0)	0.69	18.0 (9.0, 33.0)	27.0 (10.0-37.0)	0.29
Age—years												
<12 years	3,203 (0.2)	287,094 (10.9)	0.50	2,728 (0.2)	200,665 (17.3)	0.70	32,927 (41.5)	310,663 (12.1)	0.78	32,925 (41.5)	21,721 (27.4)	0.41
12-19 years	124,242 (6.7)	148,015 (5.6)		83,330 (7.2)	64,747 (5.6)		7,192 (9.1)	172,096 (6.7)		7,192 (9.1)	4,426 (5.6)	
20-29 years	391,218 (21.0)	606,809 (23.0)		247,288 (21.3)	290,299 (25.0)		12,017 (15.1)	559,873 (21.7)		12,017 (15.1)	18,895 (23.8)	
30-39 years	690,258 (37.1)	872,640 (33.0)		415,028 (35.7)	361,418 (31.1)		17,074 (21.5)	791,145 (30.7)		17,073 (21.5)	18,713 (23.6)	
40-49 years	410,963 (22.1)	461,196 (17.5)		241,989 (20.8)	165,904 (14.3)		7,663 (9.6)	457,474 (17.7)		7,663 (9.7)	9,240 (11.6)	
50-59 years	171,984 (9.2)	189,395 (7.2)		116,792 (10.0)	59,259 (5.1)		1,949 (2.5)	193,923 (7.5)		1,949 (2.5)	4,068 (5.1)	
60-69 years	53,450 (2.9)	60,290 (2.3)		42,602 (3.7)	16,205 (1.4)		452 (0.6)	69,580 (2.7)		452 (0.6)	1,742 (2.2)	
70 + years	15,460 (0.8)	16,698 (0.6)		13,205 (1.1)	4,465 (0.4)		138 (0.2)	23,321 (0.9)		138 (0.2)	604 (0.8)	
Sex												
Male	1,344,584 (72.3)	1,875,809 (71.0)	0.03	820,612 (70.6)	831,073 (71.5)	0.02	39,228 (49.4)	1,705,334 (66.1)	0.34	39,227 (49.4)	50,190 (63.2)	0.28
Female	516,194 (27.7)	766,328 (29.0)		342,350 (29.4)	331,889 (28.5)		40,184 (50.6)	872,741 (33.9)		40,182 (50.6)	29,219 (36.8)	
Nationality^§^												
Bangladeshi	213,691 (11.5)	188,750 (7.1)	0.22	126,717 (10.9)	68,226 (5.9)	0.27	5,834 (7.3)	154,984 (6.0)	0.54	5,834 (7.3)	2,102 (2.6)	0.67
Egyptian	99,002 (5.3)	144,718 (5.5)		65,654 (5.6)	65,349 (5.6)		4,574 (5.8)	138,343 (5.4)		4,574 (5.8)	3,261 (4.1)	
Filipino	170,693 (9.2)	169,605 (6.4)		102,316 (8.8)	73,086 (6.3)		14,657 (18.5)	203,054 (7.9)		14,657 (18.5)	4,576 (5.8)	
Indian	478,917 (25.7)	769,165 (29.1)		283,553 (24.4)	361,340 (31.1)		28,016 (35.3)	684,408 (26.5)		28,014 (35.3)	22,031 (27.7)	
Nepalese	179,519 (9.6)	231,832 (8.8)		99,204 (8.5)	95,998 (8.3)		2,171 (2.7)	188,253 (7.3)		2,171 (2.7)	5,036 (6.3)	
Pakistani	89,745 (4.8)	138,258 (5.2)		57,988 (5.0)	62,442 (5.4)		4,458 (5.6)	149,352 (5.8)		4,458 (5.6)	5,052 (6.4)	
Qatari	190,047 (10.2)	259,664 (9.8)		144,617 (12.4)	112,001 (9.6)		2,334 (2.9)	289,136 (11.2)		2,334 (2.9)	9,500 (12.0)	
Sri Lankan	63,032 (3.4)	74,619 (2.8)		36,096 (3.1)	26,519 (2.3)		1,882 (2.4)	85,960 (3.3)		1,882 (2.4)	2,394 (3.0)	
Sudanese	40,581 (2.2)	56,725 (2.1)		26,295 (2.3)	25,376 (2.2)		1,406 (1.8)	54,888 (2.1)		1,406 (1.8)	1,543 (1.9)	
Other nationalities^¶^	335,551 (18.0)	608,801 (23.0)		220,522 (19.0)	272,625 (23.4)		14,080 (17.7)	629,697 (24.4)		14,079 (17.7)	23,914 (30.1)	
Coexisting conditions												
None	1,525,533 (82.0)	2,262,057 (85.6)	0.11	927,465 (79.8)	1,025,057 (88.1)	0.28	67,677 (85.2)	2,204,695 (85.5)	0.18	67,674 (85.2)	71,918 (90.6)	0.17
1 condition	169,924 (9.1)	218,316 (8.3)		114,066 (9.8)	91,540 (7.9)		8,738 (11.0)	210,154 (8.2)		8,738 (11.0)	5,431 (6.8)	
2 conditions	80,950 (4.4)	84,767 (3.2)		56,322 (4.8)	28,229 (2.4)		2,184 (2.8)	84,070 (3.3)		2,184 (2.8)	1,360 (1.7)	
3 conditions	37,789 (2.0)	35,440 (1.3)		27,750 (2.4)	9,132 (0.8)		461 (0.6)	35,652 (1.4)		461 (0.6)	362 (0.5)	
4 conditions	21,717 (1.2)	19,403 (0.7)		16,903 (1.5)	4,330 (0.4)		172 (0.2)	20,050 (0.8)		172 (0.2)	143 (0.2)	
5 conditions	12,480 (0.7)	10,955 (0.4)		10,053 (0.9)	2,270 (0.2)		101 (0.1)	11,652 (0.5)		101 (0.1)	86 (0.1)	
6 + conditions	12,385 (0.7)	11,199 (0.4)		10,403 (0.9)	2,404 (0.2)		79 (0.1)	11,802 (0.5)		79 (0.1)	109 (0.1)	
Clinical vulnerability status												
Less clinically vulnerable	1,536,850 (82.6)	2,284,729 (86.5)	0.11	934,093 (80.3)	1,051,290 (90.4)	0.29	74,493 (93.8)	2,204,450 (85.5)	0.28	42,551 (53.6)	50,615 (63.7)	0.14
More clinically vulnerable	323,928 (17.4)	357,408 (13.5)		228,869 (19.7)	111,672 (9.6)		4,919 (6.2)	373,625 (14.5)		36,858 (46.4)	28,794 (36.3)	

COVID-19 denotes coronavirus disease 2019, IQR interquartile range, SARS-CoV-2 severe acute respiratory syndrome coronavirus 2, and SMD standardized mean difference.

*Persons who received their second vaccine dose in a specific calendar week in the two-dose cohort were matched exactly one-to-one to persons who had a record for a SARS-CoV-2-negative test in that same calendar week in the control cohort, to ensure that matched pairs had presence in Qatar over the same time period. An iterative selection process was implemented to ensure that controls were unvaccinated and had no documented SARS-CoV-2 infection in the 90 days prior to the start of follow-up, which was set at the date of the second vaccine dose of their match.

^†^SMD is the difference in the mean of a covariate between groups divided by the pooled standard deviation.

^‡^SMD is for the mean difference between groups divided by the pooled standard deviation.

^§^Nationalities were chosen to represent the most populous groups in Qatar.

^¶^These comprise up to 183 other nationalities in the unmatched cohorts and 181 other nationalities in the matched cohorts in the pre-omicron phase analysis, and up to 182 other nationalities in the unmatched cohorts and 167 other nationalities in the matched cohorts in the omicron phase analysis.

**Table 2:** Number needed to vaccinate to prevent A) one SARS-CoV-2 infection and B) one severe, critical, or fatal COVID-19 case during the pre-omicron phase.

	Two-dose cohort			Unvaccinated cohort			${{\overset{―}{𝐒}}_{𝐮𝐧𝐯𝐚𝐜𝐜𝐢𝐧𝐚𝐭𝐞𝐝}\left(𝐭\right)}^{AHR}$	${\overset{―}{S}}_{unvaccinated}\left(t\right)$	Number needed to vaccinate (95% CI)
	Incident infections	Total person-weeks	Incidence rate per 10,000 person-weeks	Incident infections	Total person-weeks	Incidence rate per 10,000 person-weeks			
**A) To prevent one SARS-CoV-2 infection**									
Overall	10,029	20,008,293	5.01 (4.92-5.11)	40,296	19,456,650	20.71 (20.51-20.91)	0.9920816	0.9638481	35.42 (24.38-49.94)
Age									
<50 years	7,584	16,769,026	4.52 (4.42-4.63)	38,337	18,249,484	21.01 (20.80-21.22)	0.9924472	0.9649476	36.36 (25.02-51.26)
≥50 years	2,445	3,239,268	7.55 (7.25-7.85)	1,959	1,207,167	16.23 (15.53-16.96)	0.9876319	0.9548207	30.48 (21.06-43.05)
Clinical vulnerability status									
Less clinically vulnerable	6,605	15,751,386	4.19 (4.09-4.30)	36,555	17,787,956	20.55 (20.34-20.76)	0.9932792	0.9664044	37.21 (25.56-52.38)
More clinically vulnerable	3,424	4,256,907	8.04 (7.78-8.32)	3,741	1,668,694	22.42 (21.71-23.15)	0.9868323	0.9489459	26.39 (18.32-37.46)
Age—years									
0-11 years	4	26,639	1.50 (0.56-4.00)	7,250	4,590,586	15.79 (15.43-16.16)	0.9968421	0.9652730	31.68 (21.88-44.99)
12-19 years	481	1,234,565	3.90 (3.56-4.26)	2,624	922,029	28.46 (27.39-29.57)	0.9939843	0.9673126	37.49 (26.15-53.59)
20-29 years	1,372	4,098,206	3.35 (3.18-3.53)	9,899	4,686,670	21.12 (20.71-21.54)	0.9946058	0.9679548	37.52 (25.79-53.31)
30-39 years	3,272	7,125,676	4.59 (4.44-4.75)	13,130	5,522,417	23.78 (23.37-24.19)	0.9924726	0.9639097	35.01 (24.12-48.69)
40-49 years	2,455	4,283,939	5.73 (5.51-5.96)	5,434	2,527,780	21.50 (20.93-22.08)	0.9906601	0.9619394	34.82 (23.76-49.51)
50-59 years	1,464	2,168,181	6.75 (6.42-7.11)	1,507	890,135	16.93 (16.10-17.81)	0.9888591	0.9589600	33.45 (23.19-47.26)
60-69 years	721	813,299	8.87 (8.24-9.54)	337	247,874	13.60 (12.22-15.13)	0.9851848	0.9476520	26.64 (17.89-37.53)
70 + years	260	257,788	10.09 (8.93-11.39)	115	69,158	16.63 (13.85-19.96)	0.9835904	0.9374400	21.67 (15.74-31.1)
Coexisting conditions									
None	6,153	15,722,880	3.91 (3.82-4.01)	33,408	17,264,120	19.35 (19.14-19.56)	0.9935470	0.9684376	39.83 (27.33-56.01)
1 condition	1,586	2,025,128	7.83 (7.46-8.23)	4,456	1,566,438	28.45 (27.62-29.29)	0.9889838	0.9471491	23.90 (16.45-33.80)
2 conditions	918	1,024,194	8.96 (8.40-9.56)	1,495	415,516	35.98 (34.20-37.85)	0.9866397	0.9386478	20.84 (14.4-30.08)
3 conditions	494	520,952	9.48 (8.68-10.36)	469	111,016	42.25 (38.59-46.25)	0.9856178	0.9325558	18.85 (13.16-26.32)
4 conditions	375	322,435	11.63 (10.51-12.87)	216	47,664	45.32 (39.66-51.78)	0.9804022	0.9207156	16.75 (11.27-24.2)
5 conditions	221	194,624	11.36 (9.95-12.96)	111	23,846	46.55 (38.65-56.07)	0.9817199	0.9189346	15.93 (11.15-23.36)
6 + conditions	282	198,081	14.24 (12.67-16.00)	141	28,050	50.27 (42.62-59.29)	0.9763890	0.9035165	13.72 (9.61-18.50)
**B) To prevent one severe, critical, or fatal COVID-19**									
Overall	121	20,008,293	0.06 (0.05-0.07)	779	19,456,650	0.40 (0.37-0.43)	0.9999336	0.9985408	717.98 (469.38-984.01)
Age									
<50 years	29	16,769,026	0.02 (0.01-0.02)	490	18,249,484	0.27 (0.25-0.29)	0.9999767	0.9994757	1,996.00 (1,288.48−2,692.99)
≥50 years	92	3,239,268	0.28 (0.23-0.35)	289	1,207,167	2.39 (2.13-2.69)	0.9995936	0.9908649	114.56 (76.11-165.96)
Clinical vulnerability status									
Less clinically vulnerable	15	15,751,386	0.01 (0.01-0.02)	379	17,787,956	0.21 (0.19-0.24)	0.9999829	0.9996152	2,719.66 (1,764.21−3,922.50)
More clinically vulnerable	106	4,256,907	0.25 (0.21-0.30)	400	1,668,694	2.40 (2.17-2.64)	0.9996507	0.9922775	135.63 (90.42-190.31)
Coexisting conditions									
None	21	15,722,880	0.01 (0.01-0.02)	350	17,264,120	0.20 (0.18-0.23)	0.9999802	0.9996296	2,852.23 (1,764.61−3,833.32)
1 condition	10	2,025,128	0.05 (0.03-0.09)	141	1,566,438	0.90 (0.76-1.06)	0.9999510	0.9978056	466.1 (288.37-778.57)
2 conditions	16	1,024,194	0.16 (0.10-0.26)	98	415,516	2.36 (1.93-2.87)	0.9997741	0.9942048	179.56 (113.44-274.83)
3 conditions	18	520,952	0.35 (0.22-0.55)	62	111,016	5.58 (4.35-7.16)	0.9993845	0.9876256	85.04 (56.94-128.32)
4 conditions	24	322,435	0.74 (0.50-1.11)	41	47,664	8.60 (6.33-11.68)	0.9985405	0.9793204	52.03 (34.65-80.62)
5 conditions	7	194,624	0.36 (0.17-0.75)	32	23,846	13.42 (9.49-18.98)	0.9994944	0.9766983	43.87 (28.99-77.16)
6 + conditions	25	198,081	1.26 (0.85-1.87)	55	28,050	19.61 (15.05-25.54)	0.9975930	0.9573123	24.83 (15.53-36.07)

AHR denotes adjusted hazard ratio, CI, confidence interval, and S, survival probability.

The number needed to vaccinate (NNV) to avert one SARS-CoV-2 infection (or severe COVID-19 outcomes) was subsequently calculated using the expression: $\text{1}/({{\overset{―}{\text{S}}}_{\text{unvaccinated}}(\text{t})}^{AHR}-{\overset{―}{\text{S}}}_{\text{unvaccinated}}(\text{t}))$ [53]. Estimation of the 95% CIs was done using bootstrapping methods with 100 replications for computational efficiency.

Subgroup analyses by age, clinical vulnerability status, and number of coexisting conditions (Section S1) were performed whenever the sample size permitted. Individuals less clinically vulnerable to severe COVID-19 were defined as those <50 years of age and with one or no coexisting conditions [39]. Meanwhile, individuals more clinically vulnerable to severe COVID-19 were defined as those ≥50 years of age, or <50 years of age but with ≥2 coexisting conditions [39].

#### Estimation of vaccination cost-effectiveness.

As COVID-19 cost-related information is not publicly available in Qatar, costs in this study were based on the average cost of mRNA vaccination, vaccine administration, testing, and COVID-19 hospitalization in developed countries, namely Australia and the United States of America (S3 Table in S1 File) [54,55], given the similarity in socio-economic conditions with Qatar. These costs were deemed reasonable by stakeholders in the healthcare sector. All costs in this study are expressed in 2023 US dollars [56].

Infected individuals were assumed to either recover or progress to the next infection severity state. The duration and utility weights associated with each severity state were based on available literature [54,55,57–63], and were assumed to be uniform across individuals (S3 Table in S1 File).

The total direct medical costs were determined using information on the testing frequency as well as the number, duration, and cost of hospitalization of severe, critical, or fatal COVID-19 cases in each cohort. The cost of averting one infection (or severe COVID-19 outcomes) was estimated using the product of NNV and total vaccination cost per person.

QALYs for each individual were estimated by summing the product of the utility value assigned to each health state by the duration spent in that particular state. QALYs lost due to fatal COVID-19 were estimated using lifetables for Qatar retrieved from the United Nation World Population Prospects [25], after factoring the impact of coexisting conditions on age-specific life expectancies and on quality of remaining life years following the methodology outlined by Briggs et al. [64] Coexisting conditions were assumed to increase the risk of death by 50% (standardized mortality rate (SMR)=1.5) and to reduce the quality of life by 5% for individuals with 1 comorbidity, 10% with 2 comorbidities, and 20% for ≥3 comorbidities (S3 Table in S1 File) [64].

The incremental cost-effectiveness ratio (ICER) was defined as the additional cost incurred per QALY gained. We defined the cost-effectiveness threshold using the gross domestic product (GDP) per capita following established guidelines [65–67]. Interventions were deemed very cost-effective when ICER<1 GDP per capita, cost-effective when ICER is 1–3 GDP per capita, and not cost-effective when ICER>3 GDP per capita [65–67]. Qatar’s GDP per capita was $87,661 in 2022, and this value was used in setting the cost-effectiveness threshold [68]. This GDP is close to those of Australia and the United States of America, countries from which we used some of the modeling input data, estimated at $65,099.8 and $76,329.6, respectively [69].

The benefit-cost ratio was defined as the ratio of savings in direct medical costs to primary-series vaccination cost. Since the follow-up period for each cohort was less than one year, no discount rate was applied.

#### Subgroup and sensitivity analyses.

A subgroup analysis was performed to estimate ICER for individuals more clinically vulnerable to severe COVID-19. Univariate sensitivity analyses were conducted to identify parameters that would most impact ICER. The choice of parameters and their uncertainty intervals was informed by earlier studies [54,55,64] and relevance to our data (S4 Table in S1 File). Results were illustrated using tornado diagrams. An additional analysis was conducted to estimate ICER factoring a 3% annual discount rate to QALYs lost due to fatal COVID-19. Multivariate sensitivity analyses were also implemented to estimate ICER assuming best-case (lowest costs and highest QALYs) and worst-case (highest costs and lowest QALYs) scenarios (S4 Table in S1 File).

All statistical analyses were performed using Stata/SE version 18.0 (Stata Corporation, College Station, TX, USA).

## Results

### Pre-omicron phase

#### Study population.

**Fig 1:**
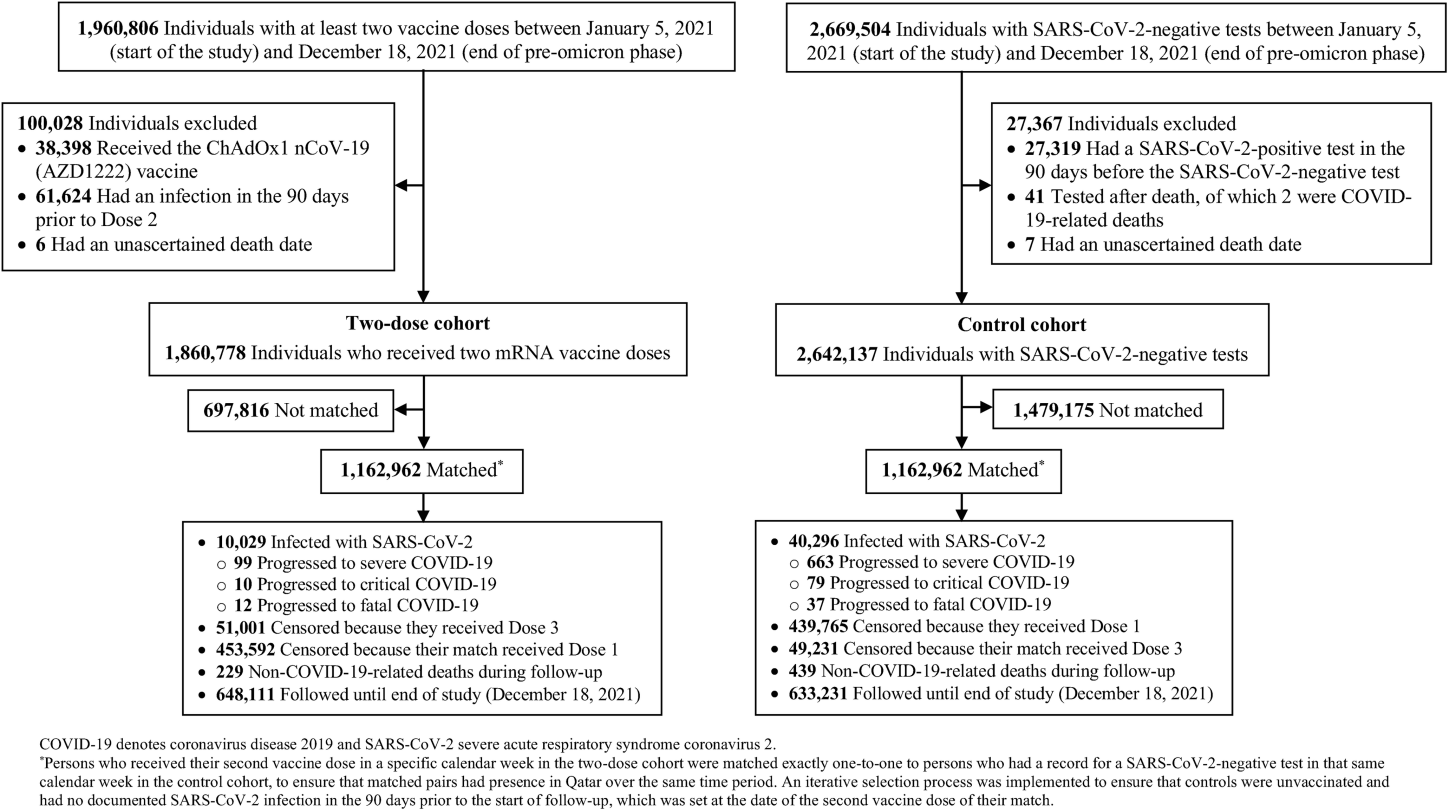
Study population selection process to estimate the number needed to vaccinate to prevent one SARS-CoV-2 infection or one severe, critical, or fatal COVID-19 case during the pre-omicron phase.

Fig 1 illustrates the study population selection process. Table 1 presents the characteristics of eligible and matched cohorts. Matched cohorts included each 1,162,962 individuals. The median follow-up duration was 111 days (interquartile range (IQR): 46–181) for the two-dose cohort and 109 days (IQR: 41–178) for the unvaccinated cohort.

#### Incidence of infection and of severe COVID-19 outcomes.

In the two-dose cohort, a total of 10,029 incident infections were documented, with 99 progressing to severe, 10 to critical, and 12 to fatal COVID-19 (Fig 1). In the unvaccinated cohort, 40,296 incident infections were documented, with 663 progressing to severe, 79 to critical, and 37 to fatal COVID-19.

After 270 days of follow-up, cumulative incidence in the two-dose cohort was 2.12% (95% CI: 2.06–2.18%) for infection (S2A Fig) and 0.03% (95% CI: 0.02–0.04%) for severe, critical, or fatal COVID-19 (S3A Fig). The corresponding estimates in the unvaccinated cohort were 4.96% (95% CI: 4.89–5.02%) for infection (S2A Fig) and 0.09% (95% CI: 0.08–0.10%) for severe, critical, or fatal COVID-19 (S3A Fig).

During follow-up, the incidence rate of infection in the two-dose cohort was 5.01 (95% CI: 4.92–5.11) per 10,000 person-weeks compared to 20.71 (95% CI: 20.51–20.91) per 10,000 person-weeks in the unvaccinated cohort (Table 2). Meanwhile, the incidence rate of severe, critical, or fatal COVID-19 was 0.06 (95% CI: 0.05–0.07) per 10,000 person-weeks in the two-dose cohort compared to 0.40 (95% CI: 0.37–0.43) per 10,000 person-weeks in the unvaccinated cohort (Table 2). Stratified incidence rates by age, clinical vulnerability status, and number of coexisting conditions are presented in Table 2.

#### Number needed to vaccinate and cost per case averted.

The estimated NNV to prevent one infection was 35.4 individuals (95% CI: 24.4–49.9; Table 2). The NNV was lower for individuals ≥50 years of age at 30.5 (95% CI: 21.1–43.1), those more clinically vulnerable to severe COVID-19 at 26.4 (95% CI: 18.3–37.5), and those with a higher number of coexisting conditions, being lowest at 13.7 (95% CI: 9.6–18.5) for individuals with ≥6 coexisting conditions.

**Fig 2:**
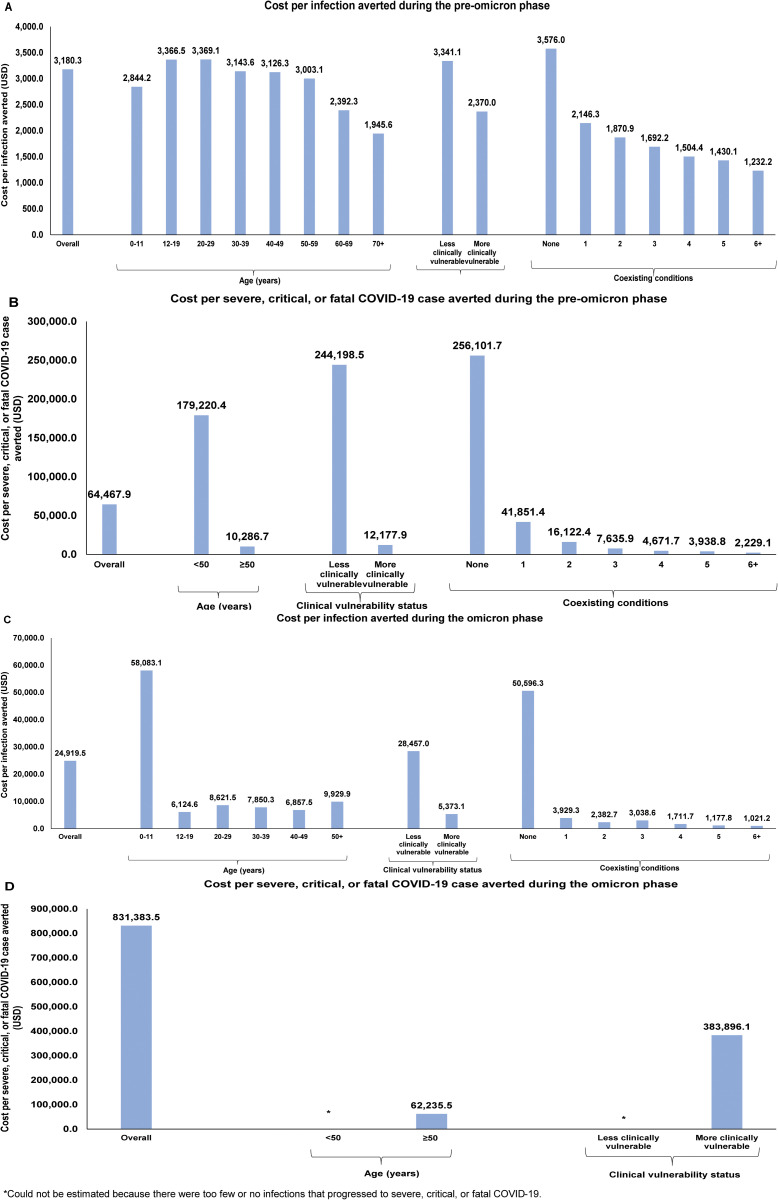
Cost per infection averted or per severe, critical, or fatal COVID-19 case averted during the pre-omicron phase (A and B, respectively) and during the omicron phase (C and D, respectively).

The overall cost per infection averted was estimated at $3,180.3 (95% CI: $2,188.9-$4,484.3; Table 3). The cost per infection averted followed the pattern observed for NNV of being lower for older age groups, those more clinically vulnerable for severe COVID-19, and those with a higher number of coexisting conditions (Fig 2A).

**Table 3:** Results of the cost-effectiveness analysis of primary-series COVID-19 vaccination in Qatar.

Measures	Two-dose cohort	Unvaccinated cohort	Incremental benefits
**Pre-omicron phase**			
Vaccination cost in US$	104,422,358.0	0	--
Incident infections	10,029	40,296	30,267
Incident severe, critical, or fatal COVID-19 cases	121	779	658
Total number of PCR tests	964,845	1,516,187	−551,342
Total number of rapid antigen tests^*^	0	0	0
Direct medical cost in US$	59,141,009.3	113,931,867.3	−54,790,858.1
Testing cost	54,677,766.2	85,922,317.3	−31,244,551.1
Hospitalization cost	4,463,243.1	28,009,550.0	−23,546,306.9
QALYs lost	114.4	839.1	724.7
ICER in US$ per QALY gained	--	--	68,485.1
Cost per infection averted in US$^†^ (95% CI)^‡^	--	--	3,180.3 (2,188.9−4,484.3)
Cost per severe, critical, or fatal COVID-19 case averted in US$^§^ (95% CI)^‡^	--	--	64,467.9 (42,145.6−88,354.2)
Benefit-cost ratio	--	--	0.52
**Omicron phase**			
Vaccination cost in US$	7,130,134.1	0	--
Incident infections	2,924	3,390	466
Incident severe, critical, or fatal COVID-19 cases	1	7	6
Total number of PCR tests	18,364	39,401	−21,037
Total number of rapid antigen tests	45,861	70,030	−24,169
Direct medical cost in US$	1,502,316.2	3,207,189.3	−1,704,873.1
Testing cost	1,471,322.7	2,890,436.4	−1,419,113.7
Hospitalization cost	30,993.5	316,752.9	−285,759.4
QALYs lost	0.0	21.2	21.2
ICER in US$ per QALY gained	--	--	255,443.6
Cost per infection averted in US$ (95% CI)^‡^	--	--	24,919.5 (24,749.9−29,112.9)
Cost per severe, critical, or fatal COVID-19 case averted in US$^§^ (95% CI)^‡^	--	--	831,383.5 (912.3−1,733,644.6)
Benefit-cost ratio	--	--	0.24

CI denotes confidence interval, ICER, incremental cost-effectiveness ratio, PCR, polymerase chain reaction, and QALY, quality-adjusted life year.

*Per national policy, SARS-CoV-2 testing during the pre-omicron phase exclusively employed PCR methodology.

^†^Cost per infection averted was calculated as the number needed to vaccinate to avert one infection multiplied by the cost of administering two vaccine doses.

^‡^95% CI calculated using the bounds of the 95% CI for the number needed to vaccinate.

^§^Cost per severe, critical, or fatal COVID-19 case averted was calculated as the number needed to vaccinate to avert one severe, critical, or fatal COVID-19 case multiplied by the cost of administering two vaccine doses.

The estimated NNV to prevent one severe, critical, or fatal COVID-19 was 718.0 individuals (95% CI: 469.4–984.0; Table 2). The NNV was lower for individuals ≥50 years of age at 114.6 (95% CI: 76.1–166.0), those more clinically vulnerable to severe COVID-19 at 135.6 (95% CI: 90.4–190.3), and those with a higher number of coexisting conditions, being lowest at 24.8 (95% CI: 15.5–36.1) for individuals with ≥6 coexisting conditions.

The overall cost per severe, critical, or fatal COVID-19 averted was estimated at $64,467.9 (95% CI: $42,145.6-$88,354.2; Table 3). The cost per severe COVID-19 outcome averted was substantially lower for individuals ≥50 years of age, those more clinically vulnerable to severe COVID-19, and those with a higher number of coexisting conditions (Fig 2B).

#### Cost-effectiveness of primary-series vaccination.

Table 3 presents the results of the cost-effectiveness analysis for primary-series vaccination during the pre-omicron phase. Primary-series vaccination incurred an additional cost of $104,422,358.0 and led to savings of $54,790,858.1 in direct medical costs, of which $23,546,306.9 were savings in hospitalization cost.

The benefit-cost ratio was 0.52, indicating that each dollar invested in primary-series vaccination reduced direct medical costs by 0.52 dollars. Primary-series vaccination led to a gain of 724.7 QALYs in the vaccinated cohort, with ICER estimated at $68,485.1 per QALY gained, indicating a very cost-effective intervention. Restricting the analysis to individuals more clinically vulnerable to severe COVID-19 yielded an ICER of $31,624.0 per QALY gained.

### Omicron phase

#### Study population.

**Fig 3:**
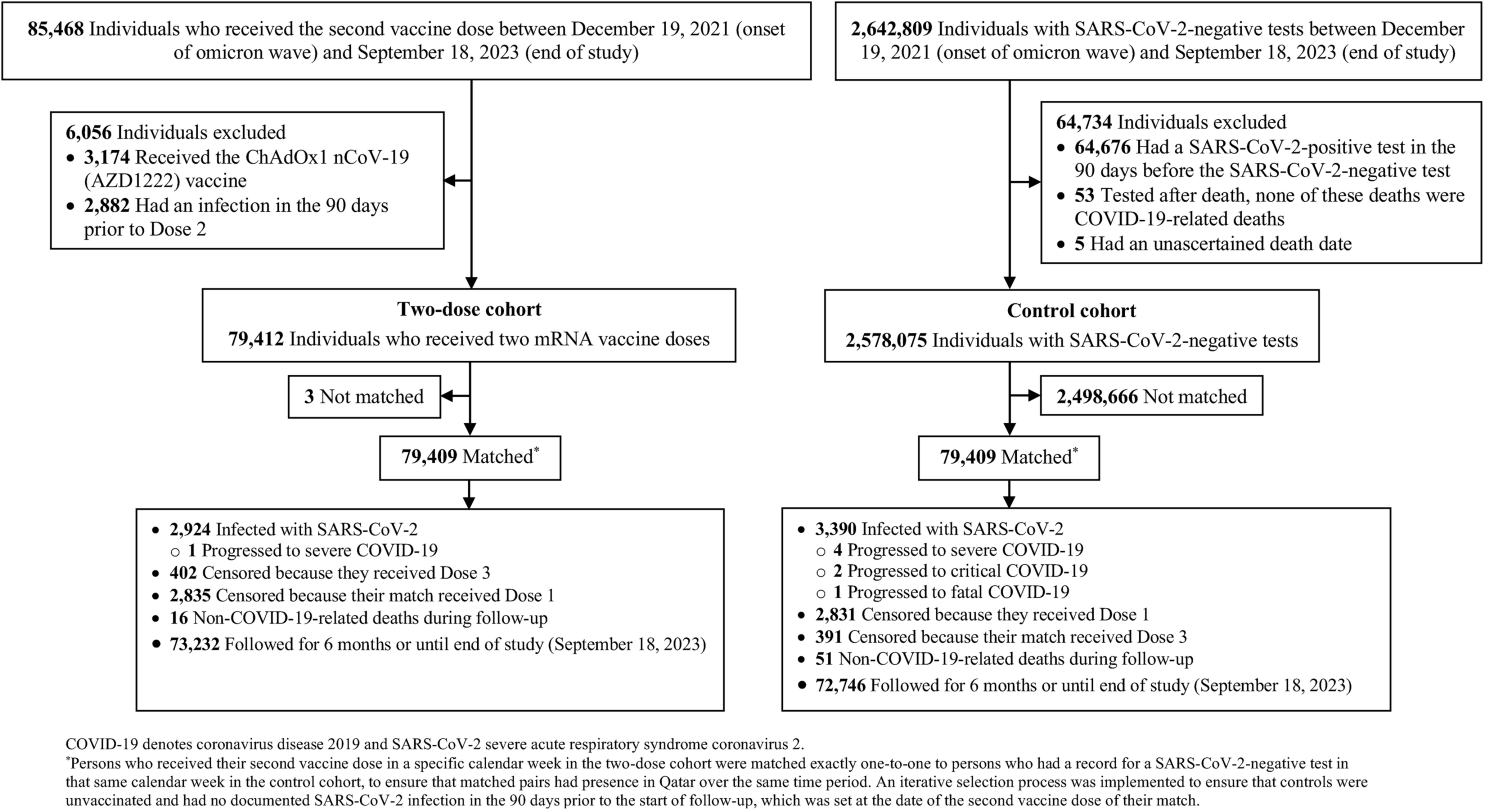
Study population selection process to estimate the number needed to vaccinate to prevent one SARS-CoV-2 infection or one severe, critical, or fatal COVID-19 case during the omicron phase.

Fig 3 illustrates the study population selection process. Table 1 presents the characteristics of eligible and matched cohorts. Matched cohorts included each 79,409 individuals. The median follow-up duration was 180 days (interquartile range (IQR): 180−180) for both the two-dose cohort and the unvaccinated cohort.

#### Incidence of infection and of severe COVID-19 outcomes.

In the two-dose cohort, a total of 2,924 incident infections were documented, with only 1 progressing to severe COVID-19 (Fig 3). In the unvaccinated cohort, 3,390 incident infections were documented, with 4 progressing to severe, 2 to critical, and 1 to fatal COVID-19.

After 180 days of follow-up, cumulative incidence in the two-dose cohort was 3.77% (95% CI: 3.64–3.90%) for infection (S2B Fig) and 0.001% (95% CI: 0.0002–0.009%) for severe, critical, or fatal COVID-19 (S3B Fig). The corresponding estimates in the unvaccinated cohort were 4.34% (95% CI: 4.20–4.49%) for infection (S2B Fig) and 0.009% (95% CI: 0.004–0.019%) for severe, critical, or fatal COVID-19 (S3B Fig).

During follow-up, the incidence rate of infection in the two-dose cohort was 14.95 (95% CI: 14.42–15.51) per 10,000 person-weeks compared to 17.51 (95% CI: 16.93–18.11) per 10,000 person-weeks in the unvaccinated cohort (Table 4). Meanwhile, the incidence rate of severe, critical, or fatal COVID-19 was 0.01 (95% CI: 0.001–0.04) per 10,000 person-weeks in the two-dose cohort compared to 0.04 (95% CI: 0.02–0.08) per 10,000 person-weeks in the unvaccinated cohort (Table 4). Stratified incidence rates by age, clinical vulnerability status, and number of coexisting medical conditions are presented in Table 4.

**Table 4:** Number needed to vaccinate to prevent A) one SARS-CoV-2 infection and B) one severe, critical, or fatal COVID-19 case during the omicron phase.

	Two-dose cohort			Unvaccinated cohort			${{\overset{―}{𝐒}}_{𝐮𝐧𝐯𝐚𝐜𝐜𝐢𝐧𝐚𝐭𝐞𝐝}\left(𝐭\right)}^{AHR}$	${\overset{―}{S}}_{unvaccinated}\left(t\right)$	Number needed to vaccinate (95% CI)
	Incident infections	Total person-weeks	Incidence rate per 10,000 person-weeks	Incident infections	Total person-weeks	Incidence rate per 10,000 person-weeks			
**A) To prevent one SARS-CoV-2 infection**									
Overall	2,924	1,955,320	14.95 (14.42-15.51)	3,390	1,935,563	17.51 (16.93-18.11)	0.9636459	0.9600427	277.53 (275.64-324.23)
Age									
<12 years	1,201	816,561	14.71 (13.90-15.56)	1,220	518,580	23.53 (22.24-24.88)	0.9573541	0.9558082	646.88 (598.21-701.49)
12-49 years	1,615	1,076,925	15.00 (14.28-15.75)	1,945	1,259,570	15.44 (14.77-16.14)	0.9715592	0.9621929	106.77 (96.76-118.56)
≥50 years	108	61,835	17.47 (14.46-21.09)	225	157,414	14.29 (12.54-16.29)	0.9721675	0.9630201	109.32 (90.97-124.11)
Clinical vulnerability status									
Less clinically vulnerable	1,534	1,043,340	14.70 (13.99-15.46)	1,840	1,245,052	14.78 (14.12-15.47)	0.9640601	0.9609048	316.93 (288.86-344.17)
More clinically vulnerable	1,390	911,980	15.24 (14.46-16.06)	1,550	690,511	22.45 (21.36-23.59)	0.9669678	0.9502567	59.84 (53.22-66.38)
Age—years									
0-11 years	1,201	816,561	14.71 (13.90-15.56)	1,220	518,580	23.53 (22.24-24.88)	0.9573541	0.9558082	646.88 (598.21-701.49)
12-19 years	394	175,109	22.50 (20.38-24.84)	341	103,141	33.06 (29.73-36.76)	0.9519642	0.9373036	68.21 (60.65-75.44)
20-29 years	404	294,628	13.71 (12.44-15.12)	601	467,593	12.85 (11.87-13.92)	0.9775155	0.9671009	96.02 (86.30-106.52)
30-39 years	566	419,203	13.50 (12.43-14.66)	644	461,849	13.94 (12.91-15.06)	0.9769129	0.9654751	87.43 (77.21-99.06)
40-49 years	251	187,985	13.35 (11.80-15.11)	359	226,987	15.82 (14.26-17.54)	0.9764693	0.9633756	76.37 (68.15-87.83)
50 + years	108	61,835	17.47 (14.46-21.09)	225	157,413	14.29 (12.54-16.29)	0.9720625	0.9630201	110.59 (92.03-125.55)
Coexisting conditions									
None	2,385	1,667,763	14.30 (13.74-14.89)	2,693	1,762,665	15.28 (14.71-15.87)	0.9649836	0.9632090	563.50 (512.13-613.57)
1 condition	371	214,712	17.28 (15.61-19.13)	463	126,187	36.69 (33.50-40.19)	0.9649514	0.9420998	43.76 (39.09-48.13)
2 conditions	115	53,248	21.60 (17.99-25.93)	154	30,990	49.69 (42.43-58.20)	0.9639066	0.9262226	26.54 (23.03-30.09)
3 conditions	33	11,162	29.56 (21.02-41.59)	36	8,276	43.5 (31.38-60.30)	0.9485400	0.9189902	33.84 (26.38-43.3)
4 conditions	11	4,088	26.91 (14.90-48.59)	17	3,108	54.70 (34.00-87.99)	0.9666298	0.9141739	19.06 (13.58-35.19)
5 conditions	3	2,501	12.00 (3.87-37.19)	13	1,864	69.74 (40.50-120.11)	0.9930202	0.9167857	13.12 (8.5-21.25)
6 + conditions	6	1,845	32.52 (14.61-72.39)	14	2,473	56.61 (33.53-95.59)	0.9863744	0.8984514	11.37 (8.44-18.01)
B) To prevent one severe, critical, or fatal COVID-19*									
Overall	1	1,955,320	0.01 (0.001-0.04)	7	1,935,563	0.04 (0.02-0.08)	0.9999906	0.9998826	9,259.2 (10.16-19,307.77)
Age									
≥50 years	1	61,835	0.16 (0.02-1.15)	5	157,414	0.32 (0.13-0.76)	0.9998683	0.9984256	693.12 (3.23−2,417.37)
Clinical vulnerability status									
More clinically vulnerable	1	911,980	0.01 (0.002-0.08)	6	690,511	0.09 (0.04-0.19)	0.9999766	0.9997427	4,275.49 (7.83-9,709.41)

AHR denotes adjusted hazard ratio, CI, confidence interval, and S, survival probability.

* Number needed to vaccinate could not be estimated for other strata because there were too few or no infections that progressed to severe, critical, or fatal COVID-19.

#### Number needed to vaccinate and cost per case averted.

The estimated NNV to prevent one infection was 277.5 individuals (95% CI: 275.6–324.2; Table 4). The NNV was substantially lower for individuals 12–49 years of age estimated at 106.8 (95% CI: 96.8–118.6), and those ≥50 years of age estimated at 109.3 (95% CI: 91.0–124.1), compared to children <12 years of age. The NNV was also substantially lower for individuals more clinically vulnerable to severe COVID-19 estimated at 59.8 (95% CI: 53.2–66.4). The NNV decreased gradually with higher number of coexisting conditions, being lowest at 11.4 (95% CI: 8.4–18.0) for individuals with ≥6 coexisting conditions.

The overall cost per infection averted was estimated at $24,919.5 (95% CI: $24,749.9-$29,112.9; Table 3). The cost per infection averted followed the pattern observed for NNV and was substantially lower for individuals ≥12 years of age compared to children (Fig 2C). Lower cost per infection averted was estimated for those more clinically vulnerable to severe COVID-19 and those with a higher number of coexisting conditions.

The estimated NNV to prevent one severe, critical, or fatal COVID-19 was 9,259.2 individuals (95% CI: 10.2–19,307.8; Table 4), but with very wide 95% CI. The small size of the omicron-phase cohort and the limited number of severe COVID-19 cases during the omicron phase resulted in wide CIs around all estimates relating to severe COVID-19 and hindered estimations for some of the subgroups. The NNV was 693.1 (95% CI: 3.2–2,417.4) for individuals ≥50 years of age and 4,275.5 (95% CI: 7.8–9,709.4) for those more clinically vulnerable to severe COVID-19.

The overall cost per severe, critical, or fatal COVID-19 averted was estimated at $831,383.5 (95% CI: $912.3-$1,733,644.6; Table 3), but with very wide 95% CI. This cost per severe COVID-19 outcome averted was substantially lower for individuals ≥50 years of age and those more clinically vulnerable to severe COVID-19 (Fig 2D).

#### Cost-effectiveness of primary-series vaccination.

Table 3 presents the results of the cost-effectiveness analysis for primary-series vaccination during the omicron phase. Primary-series vaccination incurred an additional cost of only $7,130,134.1 as most individuals completed their primary-series vaccination in the pre-omicron phase (S4 Fig). Primary-series vaccination led to savings of $1,704,873.1 in direct medical costs, of which $285,759.4 were savings in hospitalization cost.

The benefit-cost ratio was 0.24, indicating that each dollar invested in primary-series vaccination reduced direct medical costs by 0.24 dollars. Primary-series vaccination led to a gain of 21.2 QALYs in the vaccinated cohort, with ICER estimated at $255,443.6 per QALY gained, indicating a cost-effective intervention. Restricting the analysis to individuals more clinically vulnerable to severe COVID-19 yielded an ICER of $127,049.1 per QALY gained.

### Sensitivity analyses

**Fig 4:**
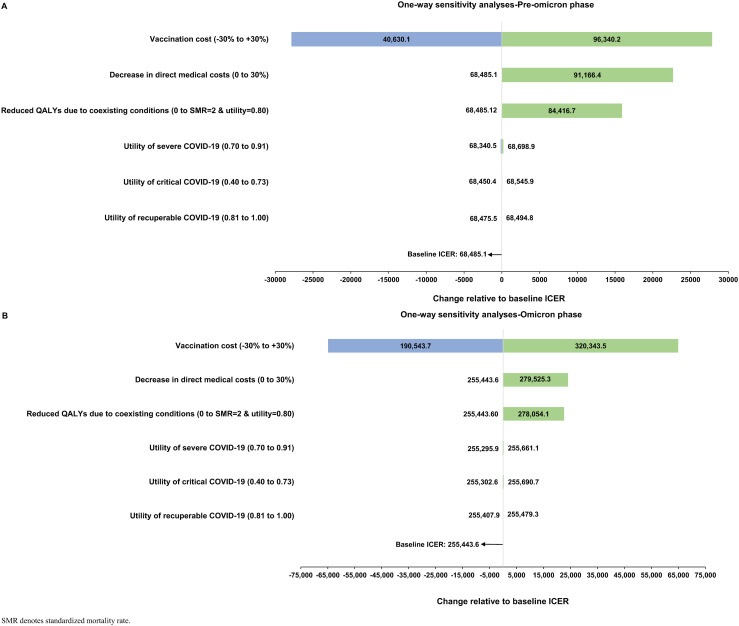
Tornado plots illustrating one-way sensitivity analyses for the A) pre-omicron phase analyses and B) omicron phase analyses. Plots show estimated values for the incremental cost-effectiveness ratios (ICERs) under different scenarios.

Fig 4 illustrates the results of one-way sensitivity analyses during the pre-omicron and omicron phases. The largest reduction in ICER during both phases was observed with reduced vaccine costs. In the pre-omicron phase, lowering vaccine costs led to an ICER of $40,630.1 per QALY gained, significantly below half of Qatar’s GDP, establishing vaccination as highly cost-effective. Every sensitivity analysis in this phase produced ICERs < 3 times the GDP per capita, indicating cost-effectiveness. However, during the omicron phase, increased vaccine costs, decreased direct medical costs, and a higher mortality risk from coexisting conditions resulted in ICERs crossing the threshold of cost-effectiveness. Implementing a 3% annual discount rate on QALYs lost to fatal COVID-19 maintained this cost-effectiveness pattern, with ICERs calculated at $100,827.8 and $354,358.3 per QALY gained for the pre-omicron and omicron phases, respectively.

The multivariate sensitivity analysis for the pre-omicron phase yielded ICERs of $40,518.1 and $147,461.8 per QALY gained for the best and worst-case scenarios, respectively—both <3 times the GDP per capita range, signifying cost-effectiveness. For the omicron phase, the best and worst-case scenarios produced ICERs of $190,302.0 and $375,712.1 per QALY gained, respectively, with the latter estimate surpassing the cost-effectiveness threshold.

## Discussion

During the pre-omicron phase, primary-series vaccination in Qatar was very cost-effective, falling below the 1 GDP per capita threshold, despite the population being young and the reduced COVID-19 severity. The vaccine played a critical role in preventing over 80% of severe, critical, and fatal instances of COVID-19, resulting in hospitalization cost savings of approximately $25 million. Even during the omicron phase, despite reduced vaccine effectiveness and lower incidence of severe cases, the vaccine still prevented the majority of severe, critical, and fatal COVID-19 outcomes. Furthermore, its cost-effectiveness remained within the 1–3 GDP per capita threshold for cost-effectiveness, demonstrating the continued value of mass vaccination.

Our findings aligned with those from other countries demonstrating the vaccination program’s cost-effectiveness, particularly during periods marked by higher infection severity and increased hospitalizations [22,54,55,70,71]. The latter finding was affirmed for the pre-omicron phase by the multivariate sensitivity analysis, assuming costs are at their highest and QALYs at their lowest. Vaccination during that period further led to substantial savings in direct medical costs, including both hospitalization and testing expenses. These findings affirm the policy decisions made to conduct mass COVID-19 vaccination as a central strategy for tackling this pandemic, with an emphasis on intensifying these efforts at times of heightened infection severity.

The NNV per averted severe, critical, or fatal COVID-19 case, and therefore the cost per case averted, were sizably lower for older age groups, individuals more clinically vulnerable to severe COVID-19, and those with a higher number of coexisting conditions, emphasizing the very high cost-effectiveness of prioritizing these groups for vaccination. While the NNV per severe COVID-19 outcome was estimated at 2,000–3,000 for young and healthy individuals during the pre-omicron phase, it was at only around 100 for those ≥50 years of age and those more clinically vulnerable to severe COVID-19. Moreover, it dropped even further, well below 100, for those with ≥3 coexisting conditions. Vaccinating individuals more clinically vulnerable to severe COVID-19 led to a 50% reduction in ICERs. This indicates that vaccination roll-out strategies prioritizing individuals at higher risk of progressing to severe forms of COVID-19 can achieve high cost-effectiveness. Notably, this approach remains relevant even when infection severity is low and vaccine effectiveness is moderate, a finding that aligns with existing literature [21,22].

Primary-series vaccination was found to be cost-effective, but not cost-saving, with ICERs being considerably higher than those reported for other countries [22,71–74], in line with expectations. Qatar’s population consists primarily of young and healthy migrants who have come to Qatar for job opportunities [26]. Notably, only 9% of Qatar’s population is 50 years or older [26]. Considering the “healthy worker effect” [75], the risk of progression to severe forms of COVID-19 upon infection is relatively low in this population [44,76]. With the cost per severe COVID-19 outcome averted hovering around $245,000 for individuals less clinically vulnerable to severe COVID-19 and those with no coexisting conditions during the pre-omicron phase (Fig 2B), mass vaccination was unlikely to be cost-saving, though it remained cost-effective, particularly during the pre-omicron phase. These findings are consistent with global evidence, demonstrating that mass primary-vaccination campaigns are cost-effective, even if they are not cost-saving [24,61,71–73,77,78].

There were large differences in cost-effectiveness between the pre-omicron and omicron phases. The ICER increased from $68,485.1 per QALY to $255,443.6, the benefit-cost ratio decreased from 0.52 to 0.24, and the cost per infection averted or severe COVID-19 outcome averted increased substantially. Several factors contribute to this finding. Firstly, first-generation COVID-19 vaccines offer only moderate protection against omicron subvariants [17–19], and this protection wanes more rapidly compared to pre-omicron variants [17–19], primarily due to inferior matching between pre-omicron immunity and omicron subvariants [39,79]. Secondly, a substantial portion of primary-series vaccinations during the omicron phase were pediatric vaccinations, which commenced only in this phase [41]; however, pediatric vaccination exhibits low effectiveness that wanes more quickly than adult vaccination [41], attributable to the lower antigen dose [40]. Thirdly, omicron subvariants demonstrate lower severity compared to pre-omicron variants [31,32,36,80–82]. Fourthly, some individuals most vulnerable to COVID-19 were infected in the pre-omicron phase and did not survive to reach the omicron phase [43]. Lastly, many individuals who received their primary-series vaccination in the omicron phase have already been infected in earlier waves (susceptible depletion) [83], substantially reducing their risk of severe COVID-19 regardless of vaccination [17,31,32,52,84–86]. Despite these factors, it is remarkable that primary-series vaccination remained cost-effective during the omicron phase, with an ICER within the 1–3 GDP per capita threshold.

Primary-series vaccination was found to be cost-effective despite our conservative approach to estimating its cost-effectiveness. Hospitalization costs were considered exclusively for severe, critical, and fatal COVID-19 cases that met the stringent WHO criteria for severity [45], criticality [45], and fatality [47]. However, other hospitalizations occurred for cases requiring care that did not reach the levels of severity outlined by the WHO criteria [26,76]. For mild or moderate infections, we did not assume loss of utility or healthcare costs in our analysis. Nonetheless, these infections are still associated with some degree of morbidity, healthcare costs (such as outpatient services and medications), and reduced quality of life. Quarantine costs were not factored into the analyses, but substantial resources were allocated in Qatar to set up and run quarantine facilities, particularly due to shared accommodations for the majority segment of the population, comprising craft and manual workers [26,44,87,88].

The benefits of vaccination were considered for only a year after the second dose in the pre-omicron phase and for six months in the omicron phase, given the relatively short duration of protection against infection [11–15,17–19]. However, these vaccines also provide prolonged protection against severe forms of infection well beyond these durations [11–15,17–19]. The analyses did not account for the benefits of vaccination against Long COVID [89–91]. Only the effects of vaccination against the acquisition of infection or severe disease were included.

However, vaccination has other beneficial effects, including lower infectivity among vaccinated individuals who become infected [92–94], as well as indirect effects by reducing the pool of infected individuals and overall transmission in the population [77,95,96]. Incorporating these broader benefits, along with costs associated with infection and quarantine, would lower the ICERs and potentially demonstrate cost-saving outcomes, even for low-risk groups.

The findings of this study provide important insights into the design and implementation of effective vaccination strategies for COVID-19 and other infectious diseases. Demonstrating that the vaccine prevented the majority of severe COVID-19 cases both before and after the emergence of omicron, our findings support the strategy of mass vaccination during subsequent waves of infection. Furthermore, our previous research confirms that booster doses significantly enhance protection against severe disease—providing an additional 75% effectiveness beyond the primary series, thus highlighting their essential role in sustaining immunity [42].

Prioritizing vaccination for older adults, individuals with multiple coexisting conditions, and those with greater clinical vulnerability has been shown to achieve the highest cost-effectiveness and impact in preventing severe disease outcomes. This targeted approach is particularly advantageous in resource-limited settings and could potentially also be applied to other infectious diseases, such as influenza and respiratory syncytial virus (RSV) infections, which disproportionately affect high-risk populations.

The substantial differences in cost-effectiveness observed between the pre-omicron and omicron phases highlight the importance of aligning vaccine formulations with circulating pathogen variants to optimize cost-effectiveness. Future vaccination efforts should prioritize the development and deployment of variant-specific vaccines to enhance their impact and effectiveness.

The findings further demonstrate the value of integrating epidemiological data with economic modeling to inform resource allocation in vaccination programs. Such approaches enable decision-makers to balance costs and health outcomes effectively, providing a robust framework for optimizing vaccination strategies. Beyond preventing severe disease, vaccination offers broader societal benefits, including reduced transmission, lowering infection rates, decreased absenteeism, and mitigation of Long COVID. The associated reduction in healthcare costs further strengthens the case for investments in mass vaccination programs and reinforce their role as a cornerstone of pandemic preparedness and public health resilience.

This study has limitations. In the absence of publicly available data on COVID-19-related costs in Qatar, our study relied on cost data from vaccination programs in other high-income countries. Similarly, utility weights were based on available literature rather than assessed locally. We lacked data on various other costs, such as those associated with protective equipment for healthcare workers, as well as the additional personnel and work hours required to support the expansion of public healthcare facilities in Qatar during the pandemic, including the establishment of field hospitals and quarantine facilities. While there may also be costs related to vaccine wastage and storage, these are likely not large in comparison to the aforementioned expenses.

The NNV per infection averted was estimated based on documented infections, but some infections may have gone undocumented, potentially affecting our estimates. Home-based rapid-antigen testing is not captured by the health information platform and therefore was not factored into our analysis. The omicron phase estimations were based on much smaller cohorts, as the vast majority of the population received primary-series vaccination in the pre-omicron phase (S4 Fig), limiting the precision of estimations and making it not possible to estimate cost-effectiveness for some of the subgroups.

As a resource-rich country [97] with an advanced healthcare system [98] and a predominantly young population [26], Qatar has experienced lower COVID-19 severity rates compared to observations elsewhere [43,76]. Consequently, the findings of this study may not be generalizable to other countries with different demographic profiles. Despite these limitations, the study has strengths. The inputs necessary for conducting the cost-effectiveness analyses were derived from real-world empirical data, capturing the vaccine impact through national epidemiologic cohort analyses implemented as part of this study. Conducted on a total-population scale, the research included a diverse population based on national backgrounds and leveraged extensive, validated databases. The insights provided into the cost-effectiveness of vaccination contribute information for shaping future pandemic preparedness plans, refining vaccination strategies, and optimizing resource allocation.

In conclusion, Qatar’s primary-series vaccination program was cost-effective, maintaining ICERs below the 1 GDP per capita threshold during the pre-omicron phase and within the 1–3 GDP per capita range during the omicron phase, despite the young population and reduced severity of infections. These findings support the strategic adoption of mass vaccination as a central approach during an epidemic and highlight the importance of aligning vaccine formulations with circulating variants. Additionally, targeted vaccination of older adults, individuals clinically vulnerable to severe COVID-19, and those with multiple coexisting conditions further enhanced cost-effectiveness, identifying these populations as priority groups for vaccination roll-out, particularly in resource-limited settings.

## Supporting information

S1 File Appendix. (DOCX)
