# Supplementary material for: Evaluating the cost-effectiveness of COVID-19 mRNA primary-series vaccination in Qatar: An integrated epidemiological and economic analysis
Source: PLoS One. 2025 Sep 26;20(9):e0331654. doi: 10.1371/journal.pone.0331654 (PMC12469089; doi:10.1371/journal.pone.0331654)
Supplement: S1 File — (DOCX) [file pone.0331654.s001.docx]

**Appendix**

**Table of Contents**

[S1 Fig. Phases of the coronavirus disease 2019 (COVID-19) pandemic in Qatar. 2](#_Toc209268451)

[Section S1. Study population and data sources 4](#_Toc209268452)

[Section S2. Laboratory methods and variant ascertainment 8](#_Toc209268453)

[**Real-time reverse-transcription polymerase chain reaction testing** 8](#_Toc209268454)

[**Rapid antigen testing** 8](#_Toc209268455)

[**Classification of infections by variant type** 9](#_Toc209268456)

[Section S3. COVID-19 severity, criticality, and fatality classification 10](#_Toc209268457)

[**Severe COVID-19** 10](#_Toc209268458)

[**Critical COVID-19** 10](#_Toc209268459)

[**Fatal COVID-19** 11](#_Toc209268460)

[Section S4. Matching of cohorts 12](#_Toc209268461)

[S1 Table. Strengthening the Reporting of Observational Studies in Epidemiology (STROBE) checklist for cohort studies. 13](#_Toc209268462)

[S2 Table. Consolidated Health Economic Evaluation Reporting Standards (CHEERS) checklist. 15](#_Toc209268463)

[S3 Table. Parameters used in analysis of cost-effectiveness of primary-series vaccination in Qatar. 17](#_Toc209268464)

[S4 Table. Uncertainty intervals used in univariate and multivariate sensitivity analyses. 19](#_Toc209268465)

[S2 Fig. Cumulative incidence of SARS-CoV-2 infection during A) the pre-omicron phase and B) the omicron phase of the pandemic in Qatar. 21](#_Toc209268466)

[S3 Fig. Cumulative incidence of severe, critical, or fatal COVID-19 during A) the pre-omicron phase and B) the omicron phase of the pandemic in Qatar. 22](#_Toc209268467)

[S4 Fig. Distribution of primary-series vaccinations over the study duration in Qatar. Percentages represent the proportion of second vaccine doses administered in a specific month out of all primary-series vaccinations during the study period. 23](#_Toc209268468)

[References 24](#_Toc209268469)

# **S1 Fig. Phases of the coronavirus disease 2019 (COVID-19) pandemic in Qatar.**

**
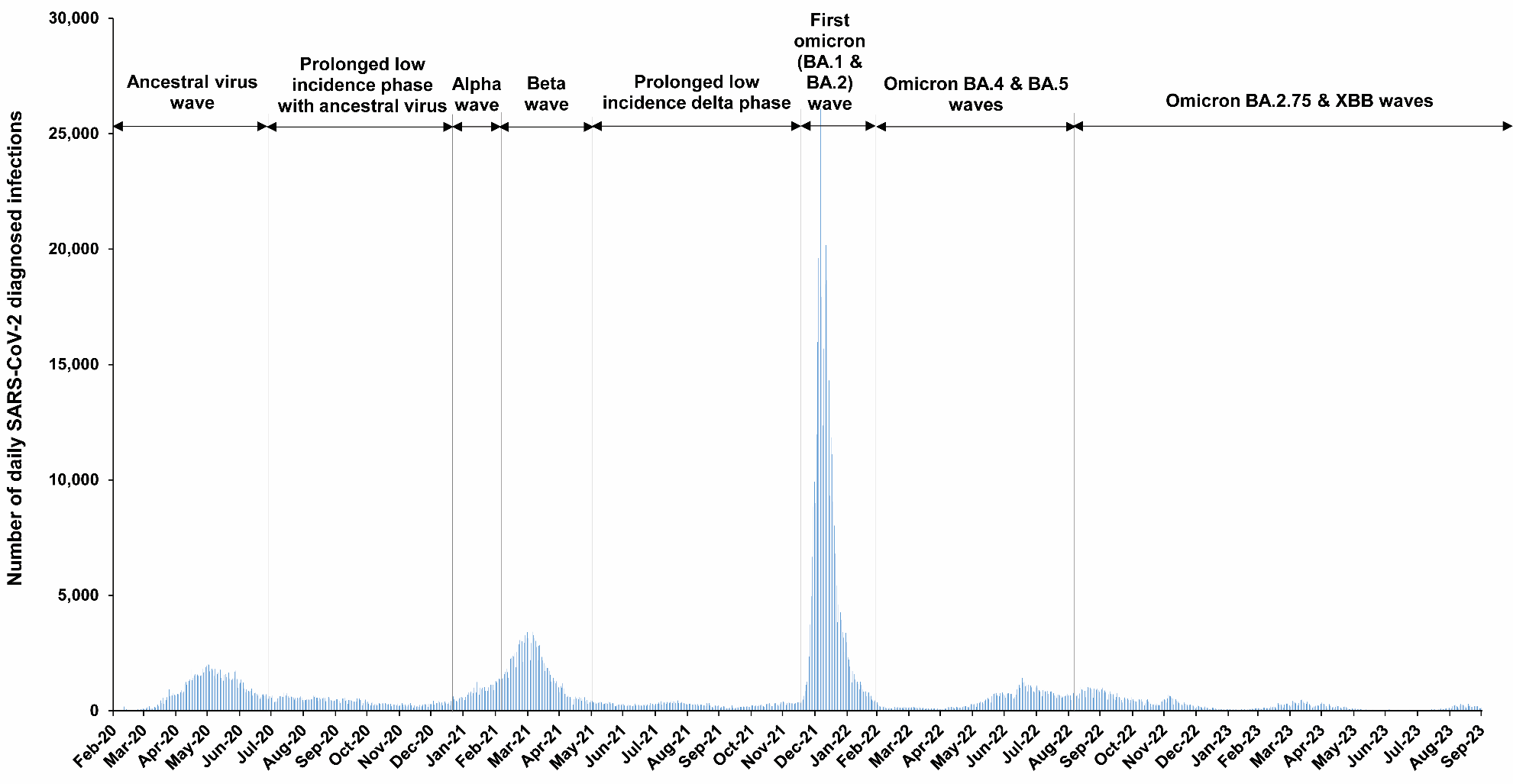
**

Phases of the coronavirus disease 2019 (COVID-19) pandemic were based on the predominant variant during that phase. These include: the ancestral virus wave (February 28, 2020 - July 31, 2020), a prolonged low incidence phase with the ancestral virus (August 1, 2020 - January 17, 2021), the alpha wave (January 18, 2021 - March 7, 2021), the beta wave (March 8, 2021 - May 31, 2021), a prolonged low incidence delta phase (June 1, 2021 - December 18, 2021), the first (BA.1 & BA.2) omicron wave (December 19, 2021 - February 28, 2022), the omicron BA.4 & BA.5 wave (March 1, 2022 - September 9, 2022), and the omicron BA.2.75 & XBB waves (September 10, 2022 – September 18, 2023).

# **Section S1. Study population and data sources**

Qatar's national and universal public healthcare system uses the Cerner-system advanced digital health platform to track all electronic health record encounters of each individual in the country, including all citizens and residents registered in the national and universal public healthcare system. Registration in the public healthcare system is mandatory for citizens and residents.

The databases analyzed in this study are data-extract downloads from the Cerner-system that have been consistently obtained on a regular (twice weekly) schedule since onset of the pandemic by the Business Intelligence Unit at Hamad Medical Corporation (HMC). HMC is the national public healthcare provider and the unique provider of coronavirus disease 2019 (COVID-19) care in Qatar. At every download, all COVID-19-related testing in medical facilities, COVID-19 vaccinations, hospitalizations related to COVID-19, and all death records regardless of cause are provided to the authors through .csv files. These databases have been analyzed throughout the pandemic not only for study-related purposes, but also to provide policymakers with summary data and analytics to inform the national response.

Every health encounter in the Cerner-system is linked to an individual through the HMC Number, which serves as a unique identifier that links all records for this individual at the national level. Databases were merged and analyzed using the HMC Number to link all records pertaining to testing, vaccinations, hospitalizations, and deaths. All deaths in Qatar are recorded by the public healthcare system. All COVID-19-related healthcare was provided exclusively in the public healthcare system. No private entity was permitted to provide COVID-19-related hospitalization. COVID-19 vaccination was also provided exclusively by the public healthcare system. These health records were tracked throughout the COVID-19 pandemic using the Cerner system. This system has been implemented in 2013, before the onset of the pandemic. This pre-established system ensured that we had access to comprehensive health records related to this study for both citizens and residents throughout the entire pandemic, allowing us to follow each person over time.

Demographic information including sex, age, and nationality for each HMC Number (individual) is registered upon issuing of the universal health card, based on the Qatar Identity Card, which is a mandatory requirement by the Ministry of Interior to every citizen and resident in the country. Data extraction from the Qatar Identity Card to the digital health platform is performed electronically through scanning techniques.

All severe acute respiratory syndrome coronavirus 2 (SARS-CoV-2) testing in any facility in Qatar is recorded in one database, the national testing database. This database covers all testing throughout the country, whether in public or private facilities. Every polymerase chain reaction (PCR) test and a proportion of the facility-based rapid antigen tests conducted in Qatar, regardless of location or setting, are classified on the basis of symptoms and the reason for testing, such as the presence of clinical symptoms, contact tracing, participation in surveys or random testing campaigns, individual requests for testing, routine healthcare testing, pre-travel requirements, at the point of entry into the country, or any other relevant reasons for testing.

Before November 1, 2022, SARS-CoV-2 testing in Qatar was performed extensively with about 5% of the population were tested every week [1]. Based on the distribution of the reason for testing up to November 1, 2022, most tests in Qatar were conducted for routine reasons, such as travel-related purposes, and about 75% of infections were diagnosed because of routine testing and not because of presence of symptoms [1, 2].

Starting from November 1, 2022, testing for SARS-CoV-2 was substantially reduced, but still close to 1% of the population are being tested every week [2]. This study factored all SARS-CoV-2-related testing included in the national testing database over the duration of follow-up.

The first omicron wave that reached its peak in January of 2022 was massive and strained the testing capacity in the country [1, 3-5]. To alleviate the burden on PCR testing, rapid antigen testing was rapidly introduced. The swift change in testing policy precluded incorporating reason for testing for a number of rapid antigen tests. While the reason for testing is documented for all PCR tests, it is not uniformly available for all rapid antigen tests.

Rapid antigen test kits are accessible for purchase at pharmacies in Qatar, but results of home-based testing are neither reported nor documented in the national databases. Since SARS-CoV-2-test outcomes were linked to specific public health measures, restrictions, and privileges, testing policy and guidelines stress facility-based testing as the core testing mechanism in the population. While facility-based testing is provided free of charge or at low subsidized costs, depending on the reason for testing, home-based rapid antigen testing is de-emphasized and not supported as part of national policy.

Coexisting conditions were ascertained and classified based on the ICD-10 codes for the conditions as recorded in the electronic health record encounters of each individual in the Cerner-system national database that includes all citizens and residents registered in the national and universal public healthcare system. The public healthcare system provides healthcare to the entire resident population of Qatar free of charge or at heavily subsidized costs, including prescription drugs. With the mass expansion of this sector in recent years, facilities have been built to cater to specific needs of subpopulations. For example, tens of facilities have been built, including clinics and hospitals, in localities with high density of craft and manual workers [6].

All encounters for each individual were analyzed to determine the coexisting-condition classification for that individual. The Cerner-system national database includes encounters starting from 2013, after this system was launched in Qatar. As long as each individual had at least one encounter with a specific coexisting-condition diagnosis since 2013, this person was classified with this coexisting condition.

Individuals who have coexisting conditions but never sought care in the public healthcare system, or seek care exclusively in private healthcare facilities, were classified as individuals with no coexisting conditions due to absence of recorded encounters for them. This misclassification bias is not likely to affect the study results. The results for those more clinically vulnerable will not be materially affected, as this misclassification bias would mainly have resulted in a smaller cohort of these persons. As for those less clinically vulnerable, the misclassification bias could imply that some of them may have been more clinically vulnerable. However, this proportion is likely to be very small compared to the proportion of those with one or no coexisting conditions in the young population of Qatar. The effect on study outcomes is thus likely to be negligible.

Further descriptions of the study population and the national databases were reported previously [1, 2, 5, 7-9].

# **Section S2. Laboratory methods and variant ascertainment**

## **Real-time reverse-transcription polymerase chain reaction testing**

Nasopharyngeal and/or oropharyngeal swabs were collected for PCR testing and placed in Universal Transport Medium (UTM). Aliquots of UTM were: 1) extracted on KingFisher Flex (Thermo Fisher Scientific, USA), MGISP-960 (MGI, China), or ExiPrep 96 Lite (Bioneer, South Korea) followed by testing with real-time reverse-transcription PCR (RT-qPCR) using TaqPath COVID-19 Combo Kits (Thermo Fisher Scientific, USA) on an ABI 7500 FAST (Thermo Fisher Scientific, USA); 2) tested directly on the Cepheid GeneXpert system using the Xpert Xpress SARS-CoV-2 (Cepheid, USA); or 3) loaded directly into a Roche cobas 6800 system and assayed with the cobas SARS-CoV-2 Test (Roche, Switzerland). The first assay targets the viral S, N, and ORF1ab gene regions. The second targets the viral N and E-gene regions, and the third targets the ORF1ab and E-gene regions.

All PCR testing was conducted at HMC Central Laboratory or Sidra Medicine Laboratory, following standardized protocols.

## **Rapid antigen testing**

SARS-CoV-2 antigen tests were performed on nasopharyngeal swabs using one of the following lateral flow antigen tests: Panbio COVID-19 Ag Rapid Test Device (Abbott, USA); SARS-CoV-2 Rapid Antigen Test (Roche, Switzerland); Standard Q COVID-19 Antigen Test (SD Biosensor, Korea); or CareStart COVID-19 Antigen Test (Access Bio, USA). All antigen tests were performed point-of-care according to each manufacturer's instructions at public or private hospitals and clinics throughout Qatar with prior authorization and training by the Ministry of Public Health (MOPH). Antigen test results were electronically reported to the MOPH in real time using the Antigen Test Management System which is integrated with the national COVID-19 database.

## **Classification of infections by variant type**

Surveillance for SARS-CoV-2 variants in Qatar is based on viral genome sequencing and multiplex RT-qPCR variant screening [10] of random positive clinical samples [10-15], complemented by deep sequencing of wastewater samples [2, 13, 16]. Further details on the viral genome sequencing and multiplex RT-qPCR variant screening throughout the SARS-CoV-2 waves in Qatar can be found in previous publications [1, 2, 4, 9, 11-15, 17-21].

# **Section S3. COVID-19 severity, criticality, and fatality classification**

Classification of COVID-19 case severity (acute-care hospitalizations) [22], criticality (intensive-care-unit hospitalizations) [23], and fatality [23] followed World Health Organization (WHO) guidelines. Assessments were made by trained medical personnel independent of study investigators and using individual chart reviews, as part of a national protocol applied to every hospitalized COVID-19 patient. Each hospitalized COVID-19 patient underwent an infection severity assessment every three days until discharge or death.

## **Severe COVID-19**

Severe COVID-19 disease was defined per WHO classification as a SARS-CoV-2 infected person with “oxygen saturation of <90% on room air, and/or respiratory rate of >30 breaths/minute in adults and children >5 years old (or ≥60 breaths/minute in children <2 months old or ≥50 breaths/minute in children 2-11 months old or ≥40 breaths/minute in children 1–5 years old), and/or signs of severe respiratory distress (accessory muscle use and inability to complete full sentences, and, in children, very severe chest wall indrawing, grunting, central cyanosis, or presence of any other general danger signs)” [24]. Detailed WHO criteria for classifying SARS-CoV-2 infection severity can be found in the WHO technical report [23].

## **Critical COVID-19**

Critical COVID-19 disease was defined per WHO classification as a SARS-CoV-2 infected person with “acute respiratory distress syndrome, sepsis, septic shock, or other conditions that would normally require the provision of life sustaining therapies such as mechanical ventilation (invasive or non-invasive) or vasopressor therapy” [23]. Detailed WHO criteria for classifying SARS-CoV-2 infection criticality can be found in the WHO technical report [23].

## **Fatal COVID-19**

COVID-19 death was defined per WHO classification as “a death resulting from a clinically compatible illness, in a probable or confirmed COVID-19 case, unless there is a clear alternative cause of death that cannot be related to COVID-19 disease (e.g. trauma). There should be no period of complete recovery from COVID-19 between illness and death. A death due to COVID-19 may not be attributed to another disease (e.g. cancer) and should be counted independently of preexisting conditions that are suspected of triggering a severe course of COVID-19”. Detailed WHO criteria for classifying COVID-19 death can be found in the WHO technical report [23].

# **Section S4. Matching of cohorts**

Each individual in the two-dose cohort was matched exactly one-to-one to a SARS-CoV-2-negative test for an individual in the control cohort who underwent testing in the same calendar week during which the individual in the two-dose cohort received the second vaccine dose. Whenever two or more SARS-CoV-2 tests for a given individual in the control cohort matched, only one of these tests was retained; the others were dropped and replaced by SARS-CoV-2 tests for controls that have not yet been matched. Accordingly, an individual in the two-dose cohort was matched to only one unique individual in the control cohort.

Matching was performed iteratively such that selected individuals from the control cohort were, at the start date of follow-up, alive, unvaccinated, and had no record of a SARS-CoV-2-positive test in the previous 90 days. The matching algorithm was implemented using *ccmatch* command in Stata supplemented with conditions to retain only controls that fulfilled these eligibility criteria and was iterated using loops with as many replications as needed until exhaustion (i.e., no more matched pairs could be identified).

Persons in the matched unvaccinated cohort contributed follow-up time before receiving primary-series vaccination (while matched to two-dose-vaccinated persons), and subsequently contributed follow-up time in the two-dose cohort, if they received primary-series vaccination (while matched to unvaccinated persons). Introducing this cross-over in the study design may reduce potential differences arising from unmeasured confounding factors related to vaccination status.

# **S1 Table. Strengthening the Reporting of Observational Studies in Epidemiology (STROBE) checklist for cohort studies.**

|  | Item No | Recommendation | Main Text page |
| --- | --- | --- | --- |
| **Title and abstract** | 1 | (*a*) Indicate the study’s design with a commonly used term in the title or the abstract | Abstract |
|  |  | (*b*) Provide in the abstract an informative and balanced summary of what was done and what was found |  |
| Introduction | | | |
| Background/rationale | 2 | Explain the scientific background and rationale for the investigation being reported | Introduction |
| Objectives | 3 | State specific objectives, including any prespecified hypotheses | Introduction |
| Methods | | | |
| Study design | 4 | Present key elements of study design early in the paper | Methods (‘Study design’) |
| Setting | 5 | Describe the setting, locations, and relevant dates, including periods of recruitment, exposure, follow-up, and data collection | Methods (‘Study population, data sources, and vaccination’ & ‘Study design’) & Sections S1-S4 in Appendix |
| Participants | 6 | (*a*) Give the eligibility criteria, and the sources and methods of selection of participants. Describe methods of follow-up | Methods (‘Study population, data sources, and vaccination’ & ‘Study design’) & Sections S1 & S4 in Appendix |
|  |  | (*b*) For matched studies, give matching criteria and number of exposed and unexposed |  |
| Variables | 7 | Clearly define all outcomes, exposures, predictors, potential confounders, and effect modifiers. Give diagnostic criteria, if applicable | Methods (‘Study design’ & ‘Statistical analysis’), & Sections S1-S3 in Appendix |
| Data sources/ measurement | 8* | For each variable of interest, give sources of data and details of methods of assessment (measurement). Describe comparability of assessment methods if there is more than one group | Methods (‘Study population, data sources, and vaccination’ & ‘Study design’), Table 1, & Sections S1-S3 in Appendix |
| Bias | 9 | Describe any efforts to address potential sources of bias | Methods (‘Statistical analysis’) |
| Study size | 10 | Explain how the study size was arrived at | Figs. 1 & 3 |
| Quantitative variables | 11 | Explain how quantitative variables were handled in the analyses. If applicable, describe which groupings were chosen and why | Methods (‘Statistical analysis’) & Table 1 & Table S3 in Appendix |
| Statistical methods | 12 | (*a*) Describe all statistical methods, including those used to control for confounding | Methods (‘Statistical analysis’) |
|  |  | (*b*) Describe any methods used to examine subgroups and interactions | Methods (‘Statistical analysis’) |
|  |  | (*c*) Explain how missing data were addressed | Not applicable, see Methods (‘Study population, data sources, and vaccination’) & Section S1 in Supplementary Information |
|  |  | (*d*) If applicable, explain how loss to follow-up was addressed | Not applicable, see Methods (‘Study population, data sources, and vaccination’) & Section S1 in Appendix |
|  |  | (*e*) Describe any sensitivity analyses | Methods (‘Subgroup and sensitivity analyses’) & Table S4 in Appendix |
| Results | | |  |
| Participants | 13* | (a) Report numbers of individuals at each stage of study—eg numbers potentially eligible, examined for eligibility, confirmed eligible, included in the study, completing follow-up, and analysed | Results (‘Study population’ in ‘Pre-omicron phase’ and in ‘Omicron phase’), Figs. 1 & 3, & Table 1 |
|  |  | (b) Give reasons for non-participation at each stage |  |
|  |  | (c) Consider use of a flow diagram |  |
| Descriptive data | 14 | (a) Give characteristics of study participants (eg demographic, clinical, social) and information on exposures and potential confounders | Results (‘Study population’ in ‘Pre-omicron phase’ and in ‘Omicron phase’), Figs. 1 & 3, & Table 1 |
|  |  | (b) Indicate number of participants with missing data for each variable of interest | Not applicable, see Methods (‘Study population, data sources, and vaccination’) & Section S1 in Appendix |
|  |  | (c) Summarise follow-up time (eg, average and total amount) | Results (‘Study population’ in ‘Pre-omicron phase’ and in ‘Omicron phase’), & Figs. S2-S3 in Appendix |
| Outcome data | 15 | Report numbers of outcome events or summary measures over time | Results (‘Incidence of infection and of severe COVID-19 outcomes’, ‘Number needed to vaccinate and cost per case averted’, & ‘Cost-effectiveness of primary-series vacciantion’ in ‘Pre-omicron phase’ and in ‘Omicron phase’), Tables 2-4, Fig. 2, & Figs. S2-S3 in Appendix |
| Main results | 16 | (a) Give unadjusted estimates and, if applicable, confounder-adjusted estimates and their precision (eg, 95% confidence interval). Make clear which confounders were adjusted for and why they were included | Results (‘Incidence of infection and of severe COVID-19 outcomes’, ‘Number needed to vaccinate and cost per case averted’, & ‘Cost-effectiveness of primary-series vacciantion’ in ‘Pre-omicron phase’ and in ‘Omicron phase’), Tables 2-4, Fig. 2, & Figs. S2-S3 in Appendix |
|  |  | (b) Report category boundaries when continuous variables were categorized | Table 1 |
|  |  | (c) If relevant, consider translating estimates of relative risk into absolute risk for a meaningful time period | Not applicable |
| Other analyses | 17 | Report other analyses done—eg analyses of subgroups and interactions, and sensitivity analyses | Results (‘Number needed to vaccinate and cost per case averted’ & ‘Cost-effectiveness of primary-series vaccination’, in ‘Pre-omicron phase’ and in ‘Omicron phase’ & ‘Sensitivity analyses’), Tables 2 & 4, & Figs. 2 & 4 |
| Discussion | | | |
| Key results | 18 | Summarise key results with reference to study objectives | Discussion, paragraphs 1-14 |
| Limitations | 19 | Discuss limitations of the study, taking into account sources of potential bias or imprecision. Discuss both direction and magnitude of any potential bias | Discussion, paragraphs 15-17 |
| Interpretation | 20 | Give a cautious overall interpretation of results considering objectives, limitations, multiplicity of analyses, results from similar studies, and other relevant evidence | Discussion, paragraph 18 |
| Generalisability | 21 | Discuss the generalisability (external validity) of the study results | Discussion, paragraph 17 |
| Other information | | | |
| Funding | 22 | Give the source of funding and the role of the funders for the present study and, if applicable, for the original study on which the present article is based | Funding |

# **S2 Table. Consolidated Health Economic Evaluation Reporting Standards (CHEERS) checklist.**

|  | **Item** | **Guidance for Reporting** | **Reported in section** |
| --- | --- | --- | --- |
| **TITLE** | | | |
| Title | 1 | Identify the study as an economic evaluation and specify the interventions being compared. | Title |
| **ABSTRACT** | | | |
| Abstract | 2 | Provide a structured summary that highlights context, key methods, results and alternative analyses. | Abstract |
| **INTRODUCTION** | | |  |
| Background and objectives | 3 | Give the context for the study, the study question and its practical relevance for decision making in policy or practice. | Introduction |
| **METHODS** | | | |
| Health economic analysis plan | 4 | Indicate whether a health economic analysis plan was developed and where available. | Methods (‘Statistical analysis’) |
| Study population | 5 | Describe characteristics of the study population (such as age range, demographics, socioeconomic, or clinical characteristics). | Methods (‘Statistical analysis’) |
| Setting and location | 6 | Provide relevant contextual information that may influence findings. | Methods (‘Study population, data sources, and vaccination’ & ‘Study design’) & Section S1 in Appendix |
| Comparators | 7 | Describe the interventions or strategies being compared and why chosen. | Methods (‘Study population, data sources, and vaccination’ & ‘Study design’) & Section S1 in Appendix |
| Perspective | 8 | State the perspective(s) adopted by the study and why chosen. | Methods (Study population, data sources, and vaccination’) |
| Time horizon | 9 | State the time horizon for the study and why appropriate. | Methods (‘Study design’) |
| Discount rate | 10 | Report the discount rate(s) and reason chosen. | Methods (‘Estimation of vaccination cost-effectiveness’ in ‘Statistical analysis’) |
| Selection of outcomes | 11 | Describe what outcomes were used as the measure(s) of benefit(s) and harm(s). | Methods (‘Estimation of vaccination cost-effectiveness’ in ‘Statistical analysis’) |
| Measurement of outcomes | 12 | Describe how outcomes used to capture benefit(s) and harm(s) were measured. | Methods (‘Estimation of vaccination cost-effectiveness’ in ‘Statistical analysis’) & Table S3 in Appendix |
| Valuation of outcomes | 13 | Describe the population and methods used to measure and value outcomes. | Methods & Sections S1-S4 & Table S3 in Appendix |
| Measurement and valuation of resources  and costs | 14 | Describe how costs were valued. | Methods (‘Estimation of vaccination cost-effectiveness’ in ‘Statistical analysis’) & Table S3 in Appendix |
| Currency, price date, and conversion | 15 | Report the dates of the estimated resource quantities and unit costs, plus the currency and year of conversion. | Methods (‘Estimation of vaccination cost-effectiveness’ in ‘Statistical analysis’) & Table S3 in Appendix |
| Rationale and  description of model | 16 | If modelling is used, describe in detail and why used. Report if the model is publicly available and where it can be accessed. | Not applicable |
| Analytics and assumptions | 17 | Describe any methods for analysing or statistically transforming data, any extrapolation methods, and approaches for validating any model used. | Methods (‘Statistical analysis’) |
| Characterizing heterogeneity | 18 | Describe any methods used for estimating how the results of the study vary for sub-groups. | Methods (‘Estimation of the number needed to vaccinate’ in ‘Statistical analysis’) |
| Characterizing  distributional effects | 19 | Describe how impacts are distributed across different individuals  or adjustments made to reflect priority populations. | Methods (‘Estimation of the number needed to vaccinate’ in ‘Statistical analysis’) |
| Characterizing uncertainty | 20 | Describe methods to characterize any sources of uncertainty in the analysis. | Methods (‘Statistical analysis’) & Table S4 in Appendix |
| Approach to engagement with patients and others affected by the study | 21 | Describe any approaches to engage patients or service recipients, the general public, communities, or stakeholders (e.g., clinicians or payers) in the design of the study. | Not applicable |
| **RESULTS** | | | |
| Study parameters | 22 | Report all analytic inputs (e.g., values, ranges, references) including uncertainty or distributional assumptions. | Results (‘Study population’ & ‘Incidence of infection and of severe COVID-19 outcomes’ in ‘Pre-omicron phase’ and in ‘Omicron phase’) & Figs. 1 & 3 & Table S3 in Appendix |
| Summary of main results | 23 | Report the mean values for the main categories of costs and outcomes of interest and summarise them in the most appropriate overall measure. | Results (‘Number needed to vaccinate and cost per case averted’, & ‘Cost-effectiveness of primary-series vaccination’ in ‘Pre-omicron phase’ and in ‘Omicron phase’), Tables 2-4, Fig. 2, & Figs. S2-S3 in Appendix |
| Effect of uncertainty | 24 | Describe how uncertainty about analytic judgments, inputs, or projections affect findings. Report the effect of choice of discount rate and time horizon, if applicable. | Results (‘Incidence of infection and of severe COVID-19 outcomes’, ‘Number needed to vaccinate and cost per case averted’, & ‘Cost-effectiveness of primary-series vaccination’ in ‘Pre-omicron phase’ and in ‘Omicron phase’ & ‘Subgroup & sensitivity analyses’), Tables 2-4, & Figs. 2 & 4 |
| Effect of engagement with patients and others affected by the study | 25 | Report on any difference patient/service recipient, general public, community, or stakeholder involvement made to the approach or findings of the study | Not applicable |
| **DISCUSSION** | | |  |
| Study findings, imitations, generalizability, and current knowledge | 26 | Report key findings, limitations, ethical or equity considerations not captured, and how these could impact patients, policy, or practice. | Discussion |
| **OTHER RELEVANT INFORMATION** | | | |
| Source of funding | 27 | Describe how the study was funded and any role of the funder in the identification, design, conduct, and reporting of the analysis | Funding |
| Conflicts of interest | 28 | Report authors conflicts of interest according to journal or  International Committee of Medical Journal Editors requirements. | Competing interests |

# **S3 Table. Parameters used in analysis of cost-effectiveness of primary-series vaccination in Qatar.**

| **Parameter** | **Value** | **Justification** | **Source** |
| --- | --- | --- | --- |
| **Vaccination cost^*^** |  |  |  |
| Vaccine cost per dose (USD) | 28.9 | Plausible value based on average vaccine cost in Australia and USA | [25, 26] |
| Vaccine administration cost (USD) | 15.9 | Plausible value based on average vaccine administration cost in Australia and USA | [25, 26] |
| **Testing cost^*^** |  |  |  |
| PCR test cost (USD) | 56.7 | Plausible value based on average test cost in Australia and USA | [25, 26] |
| RA test cost (USD) | 9.4 | Test cost in Australia | [25] |
| **Severity state** |  |  |  |
| Asymptomatic or mild/moderate infection |  |  |  |
| Duration of asymptomatic infection (days) | 6 | Estimate based on available studies | [25-29] |
| Duration of mild/moderate infection (days) | 10 | Estimate based on available studies | [25-29] |
| Cost of mild/moderate infection (USD) | 0 | Plausible value | [25, 26] |
| Average utility weight for asymptomatic or mild/moderate infection | 1 | Estimate based on available studies | [25, 26, 30-33] |
| Severe illness |  |  |  |
| Duration of mild/moderate infection (days) | 6.5 | Estimate based on available studies | [25-29] |
| Duration of severe illness (days) | 10.5 | Estimate based on available studies | [25-29] |
| Cost of hospitalization in acute-care bed (USD)^*^ | 2,951.8 | Plausible value based on average cost of hospitalization in acute-care beds in Australia and USA | [25, 26] |
| Total cost associated with severe illness (USD) | 30,993.5 | Σ (Duration in severity state × cost) = (6.5×0) + (10.5×2,951.8) | [25, 26] |
| Average utility weight for severe infection | 0.785 | Estimate based on available studies | [25, 26, 30-33] |
| Critical illness requiring ICU hospitalization with recovery |  |  |  |
| Duration of mild/moderate infection (days) | 3 | Estimate based on available studies | [25-29] |
| Duration of severe illness (days) | 7.1 | Estimate based on available studies | [25-29] |
| Duration of critical illness (days) | 11.9 | Estimate based on available studies | [25-29] |
| Duration of recuperable state (days) | 5.7 | Estimate based on available studies | [25-29] |
| Cost of hospitalization ICU-care bed (USD)^*^ | 3,412.8 | Plausible value based on average cost of hospitalization in ICU-care beds in Australia and USA | [25, 26] |
| Cost of hospitalization during recuperable state (USD)^*^ | 707.8 | Plausible value based on average cost of hospitalization during recuperable state in Australia and USA | [25, 26] |
| Total cost associated with critical illness (USD) | 65,604.5 | Σ (Duration in severity state × cost) = (3×0) + (7.1×2,951.8) + (11.9×3,412.8) + (5.7×707.8) | [25, 26] |
| Average utility weight for critical infection | 0.520 | Estimate based on available studies | [25, 26, 30-33] |
| Average utility weight during recuperable state | 0.905 | Estimate based on available studies | [25, 26, 30-33] |
| Critical illness requiring ICU hospitalization with death |  |  |  |
| Duration of mild/moderate infection (days) | 3 | Estimate based on available studies | [25-29] |
| Duration of severe illness (days) | 7.1 | Estimate based on available studies | [25-29] |
| Duration of critical illness (days) | 11.9 | Estimate based on available studies | [25-29] |
| Cost of hospitalization ICU-care bed (USD)^*^ | 3,412.8 | Plausible value based on average cost of hospitalization in ICU-care beds in Australia and USA | [25, 26] |
| Total cost associated with critical illness (USD) | 61,569.9 | Σ (Duration in severity state × cost) = (3×0) + (7.1×2,951.8) + (11.9×3,412.8) | [25, 26] |
| Average utility weight for critical infection | 0.520 | Estimate based on available studies | [25, 26, 30-33] |

ICU denotes intensive-care unit, PCR, polymerase chain reaction, RA, rapid antigen test.

^*^All costs have been expressed in 2023 US dollars using The Campbell and Cochrane Economics Methods Group (CCEMG) and the Evidence for Policy and Practice Information Center Cost conversion tool.[34]

# **S4 Table. Uncertainty intervals used in univariate and multivariate sensitivity analyses.**

| **Parameter** | **Uncertainty range** | **Justification** | **Source** |
| --- | --- | --- | --- |
| Vaccination cost | +30% | Plausible uncertainty range considered in other studies | [25, 26] |
| Decrease in direct medical cost | 0-30% | Plausible uncertainty range considered in other studies | [25, 26] |
| Utility weight for severe infection | 0.70-0.91 | Range based on available studies | [25, 26, 30-33] |
| Utility weight for critical infection | 0.40-0.73 | Range based on available studies | [25, 26, 30-33] |
| Utility weight during recuperable state after critical infection | 0.81-1.00 | Range based on available studies | [25, 26, 30-33] |
| Standardized mortality rate | 1.5 | Plausible value considering that >75% of individuals who were SARS-CoV-2 infected in our population had no documented coexisting conditions | [35] |
| Utility weight for living with comorbidities |  |  |  |
| 0 comorbidities | 1.00 | Plausible value considering existing studies | [35] |
| 1 comorbidity | 0.95 | Plausible value considering existing studies | [35] |
| 2 comorbidities | 0.90 | Plausible value considering existing studies | [35] |
| ≥3 comorbidities | 0.80 | Plausible value considering existing studies | [35] |
| Best-case scenario (simultaneously assumes all parameters below) |  |  |  |
| Vaccination cost | -30% | Expected to decrease incremental cost by decreasing cost for the vaccinated cohort | [25, 26] |
| Decrease in direct medical cost | 0% | Plausible value considering existing studies | [25, 26] |
| Utility weight for severe infection | 0.70 | Expected to increase the incremental utility by increasing the difference between the unvaccinated cohort and the vaccinated cohort | [25, 26, 30-33] |
| Utility weight for critical infection | 0.40 | Expected to increase the incremental utility by increasing the difference between the unvaccinated cohort and the vaccinated cohort | [25, 26, 30-33] |
| Utility weight during recuperable state after critical infection | 0.81 | Expected to increase the incremental utility by increasing the difference between the unvaccinated cohort and the vaccinated cohort | [25, 26, 30-33] |
| Standardized mortality rate | 1.5 | Plausible value considering that >75% of individuals who were SARS-CoV-2 infected in our population had no documented coexisting conditions | [35] |
| Utility weight for living with comorbidities |  |  | [35] |
| 0 comorbidities | 1.00 | Plausible value considering existing studies | [35] |
| 1 comorbidity | 0.95 | Plausible value considering existing studies | [35] |
| 2 comorbidities | 0.90 | Plausible value considering existing studies | [35] |
| ≥3 comorbidities | 0.80 | Plausible value considering existing studies | [35] |
| Worst-case scenario (simultaneously assumes all parameters below) |  |  |  |
| Vaccination cost | +30% | Expected to increase incremental cost by increasing cost for the vaccinated cohort | [25, 26] |
| Decrease in direct medical cost | 30% | Expected to increase incremental cost because the relative reduction in direct medical cost is higher for the unvaccinated cohort compared to the vaccinated cohort for whom the vaccination cost will also need to be factored | [25, 26] |
| Utility weight for severe infection | 0.91 | Expected to reduce the incremental utility by reducing the difference between the unvaccinated cohort and the vaccinated cohort | [25, 26, 30-33] |
| Utility weight for critical infection | 0.73 | Expected to reduce the incremental utility by reducing the difference between the unvaccinated cohort and the vaccinated cohort | [25, 26, 30-33] |
| Utility weight during recuperable state after critical infection | 1.00 | Expected to reduce the incremental utility by reducing the difference between the unvaccinated cohort and the vaccinated cohort | [25, 26, 30-33] |
| Standardized mortality rate | 2.00 | Plausible value based on available studies, expected to lower life expectancy for individuals with comorbidities and therefore to reduce the incremental utility | [35] |
| Utility weight for living with comorbidities | 0.80 | Assumed for all individuals regardless of the number of comorbidities, expected to reduce the incremental utility | [35] |

# **S2 Fig. Cumulative incidence of SARS-CoV-2 infection during A) the pre-omicron phase and B) the omicron phase of the pandemic in Qatar.**


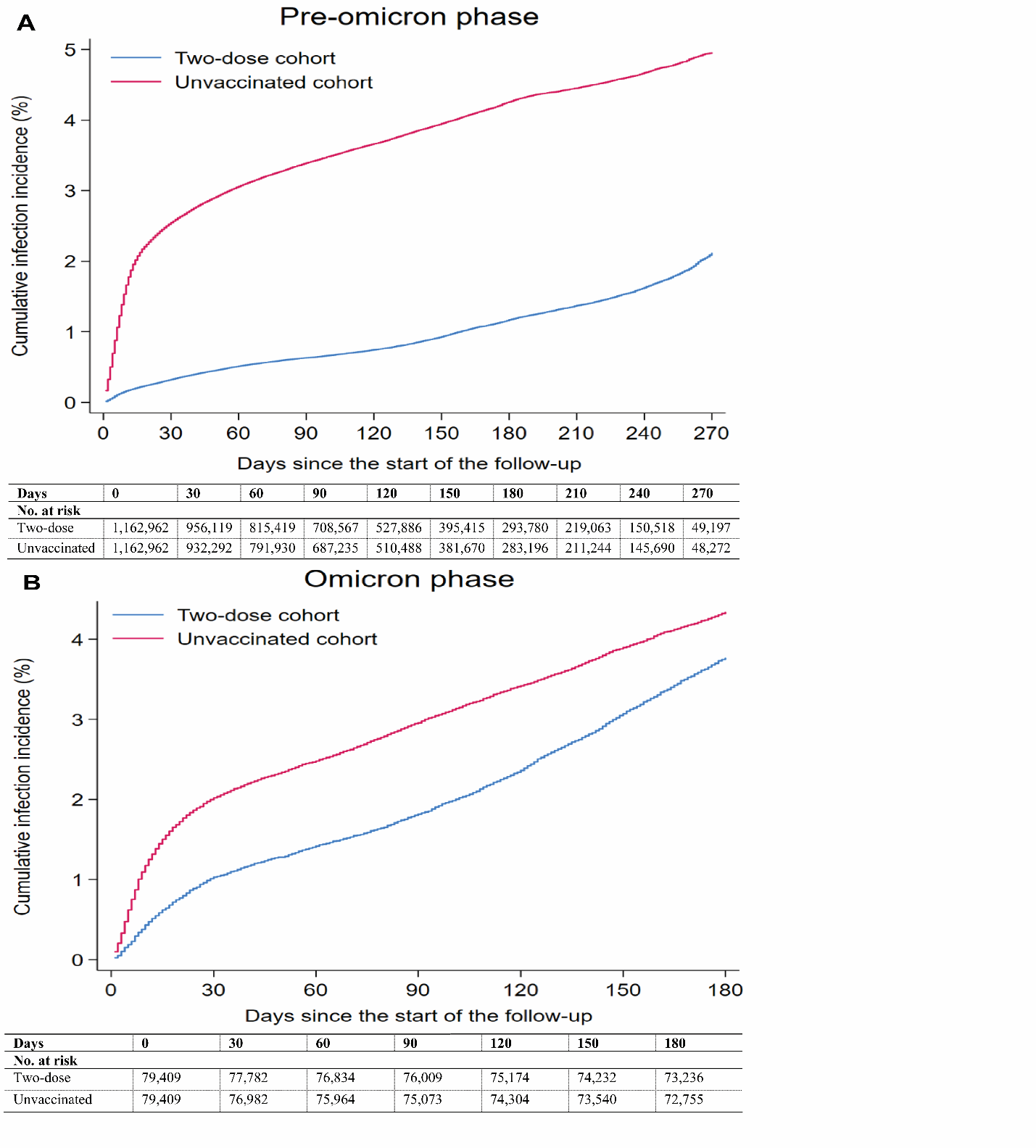


# **S3 Fig. Cumulative incidence of severe, critical, or fatal COVID-19 during A) the pre-omicron phase and B) the omicron phase of the pandemic in Qatar.**

**
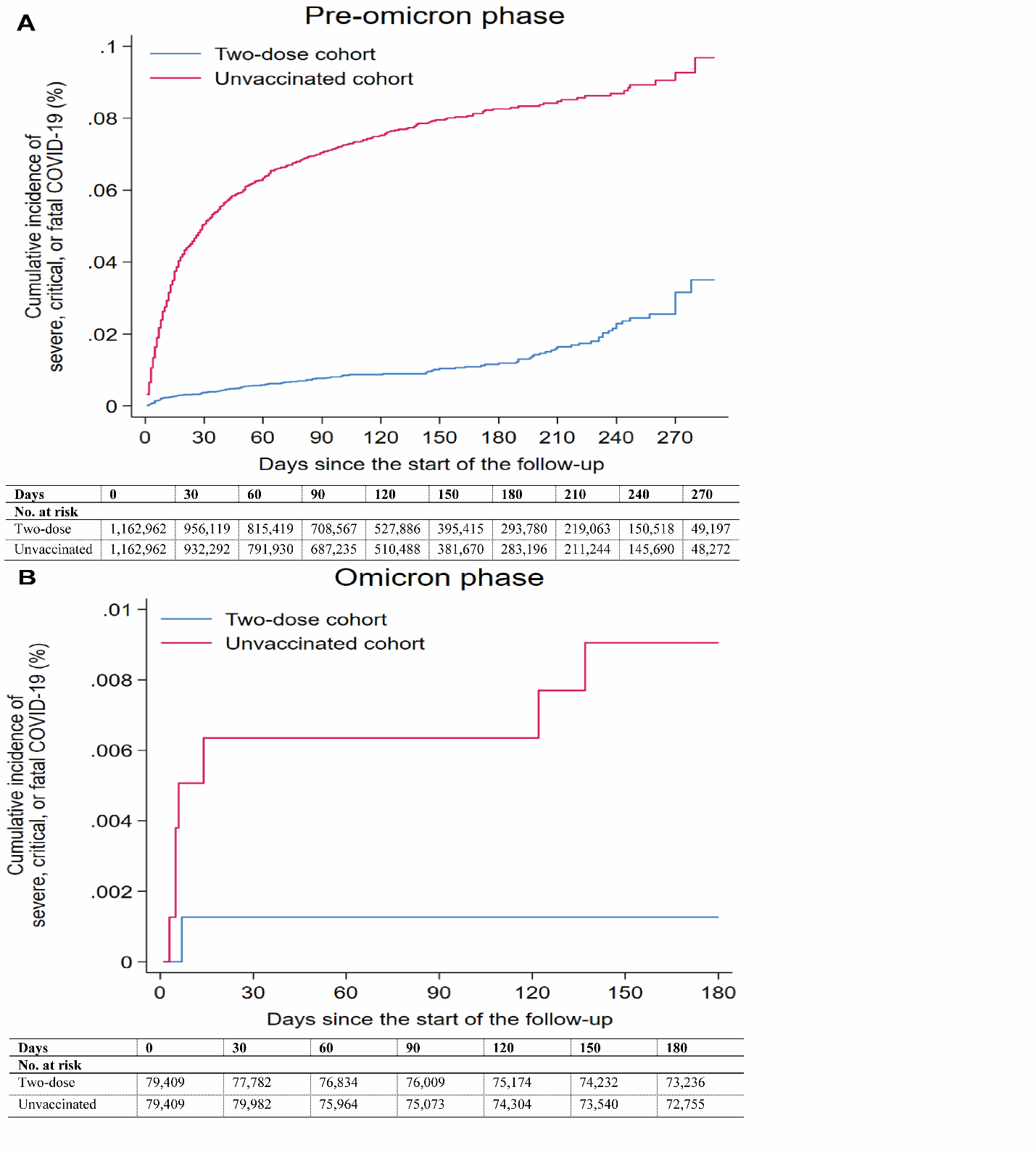
**

# **S4 Fig. Distribution of primary-series vaccinations over the study duration in Qatar. Percentages represent the proportion of second vaccine doses administered in a specific month out of all primary-series vaccinations during the study period.**

**
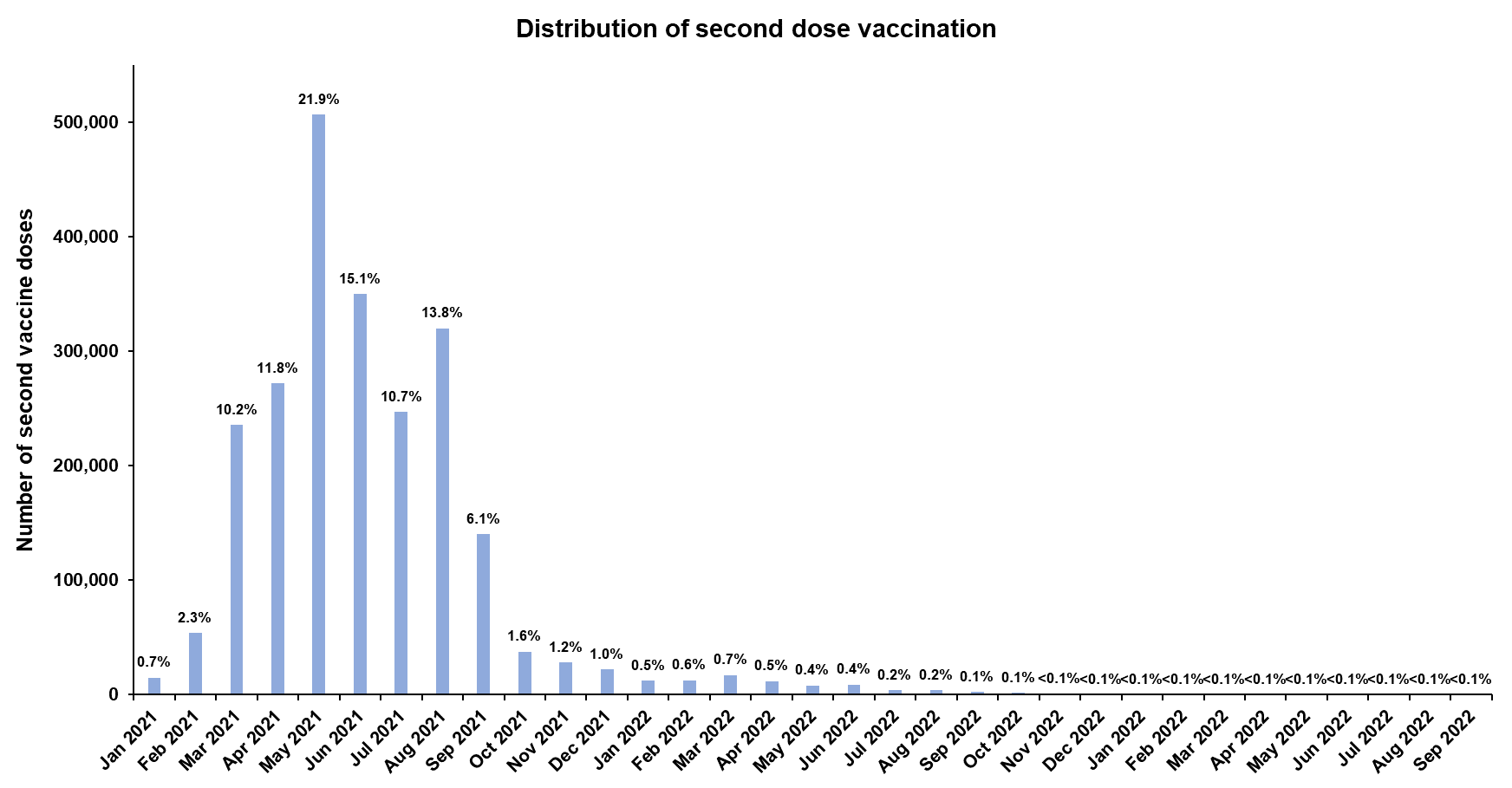
**

# **References**

1. Altarawneh HN, Chemaitelly H, Ayoub HH, Tang P, Hasan MR, Yassine HM, et al. Effects of previous infection and vaccination on symptomatic Omicron infections. The New England journal of medicine. 2022;387(1):21-34. Epub 2022/06/16. doi: 10.1056/NEJMoa2203965. PubMed PMID: 35704396; PubMed Central PMCID: PMCPMC9258753.

2. Chemaitelly H, Tang P, Hasan MR, AlMukdad S, Yassine HM, Benslimane FM, et al. Waning of BNT162b2 vaccine protection against SARS-CoV-2 infection in Qatar. The New England journal of medicine. 2021;385(24):e83. Epub 2021/10/07. doi: 10.1056/NEJMoa2114114. PubMed PMID: 34614327; PubMed Central PMCID: PMCPMC8522799.

3. Chemaitelly H, Ayoub HH, AlMukdad S, Faust JS, Tang P, Coyle P, et al. Bivalent mRNA-1273.214 vaccine effectiveness against SARS-CoV-2 omicron XBB* infections. J Travel Med. 2023;30(5). Epub 2023/08/09. doi: 10.1093/jtm/taad106. PubMed PMID: 37555656; PubMed Central PMCID: PMCPMC10481416.

4. Altarawneh HN, Chemaitelly H, Hasan MR, Ayoub HH, Qassim S, AlMukdad S, et al. Protection against the Omicron variant from previous SARS-CoV-2 infection. The New England journal of medicine. 2022;386(13):1288-90. Epub 2022/02/10. doi: 10.1056/NEJMc2200133. PubMed PMID: 35139269; PubMed Central PMCID: PMCPMC8849180.

5. Chemaitelly H, Ayoub HH, Tang P, Coyle P, Yassine HM, Al Thani AA, et al. Long-term COVID-19 booster effectiveness by infection history and clinical vulnerability and immune imprinting: a retrospective population-based cohort study. The Lancet Infectious diseases. 2023;23(7):816-27. Epub 2023/03/14. doi: 10.1016/S1473-3099(23)00058-0. PubMed PMID: 36913963; PubMed Central PMCID: PMCPMC10079373 Sciences unrelated to the work presented in this paper. All other authors declare no competing interests.

6. Al-Thani MH, Farag E, Bertollini R, Al Romaihi HE, Abdeen S, Abdelkarim A, et al. SARS-CoV-2 Infection Is at Herd Immunity in the Majority Segment of the Population of Qatar. Open Forum Infect Dis. 2021;8(8):ofab221. Epub 2021/08/31. doi: 10.1093/ofid/ofab221. PubMed PMID: 34458388; PubMed Central PMCID: PMCPMC8135898.

7. Abu-Raddad LJ, Chemaitelly H, Ayoub HH, Al Kanaani Z, Al Khal A, Al Kuwari E, et al. Characterizing the Qatar advanced-phase SARS-CoV-2 epidemic. Sci Rep. 2021;11(1):6233. Epub 2021/03/20. doi: 10.1038/s41598-021-85428-7. PubMed PMID: 33737535; PubMed Central PMCID: PMCPMC7973743.

8. Chemaitelly H, Bertollini R, Abu-Raddad LJ, National Study Group for Covid Epidemiology. Efficacy of Natural Immunity against SARS-CoV-2 Reinfection with the Beta Variant. N Engl J Med. 2021;385(27):2585-6. Epub 2021/12/16. doi: 10.1056/NEJMc2110300. PubMed PMID: 34910864; PubMed Central PMCID: PMCPMC8693689.

9. Abu-Raddad LJ, Chemaitelly H, Ayoub HH, AlMukdad S, Yassine HM, Al-Khatib HA, et al. Effect of mRNA vaccine boosters against SARS-CoV-2 Omicron infection in Qatar. The New England journal of medicine. 2022;386(19):1804-16. Epub 2022/03/10. doi: 10.1056/NEJMoa2200797. PubMed PMID: 35263534; PubMed Central PMCID: PMCPMC8929389.

10. Vogels C, Fauver J, Grubaugh N. Multiplexed RT-qPCR to screen for SARS-COV-2 B.1.1.7, B.1.351, and P.1 variants of concern V.3. dx.doi.org/10.17504/protocols.io.br9vm966. 2021;(June 6, 2021).

11. Abu-Raddad LJ, Chemaitelly H, Butt AA, National Study Group for Covid Vaccination. Effectiveness of the BNT162b2 Covid-19 Vaccine against the B.1.1.7 and B.1.351 Variants. N Engl J Med. 2021;385(2):187-9. Epub 2021/05/06. doi: 10.1056/NEJMc2104974. PubMed PMID: 33951357; PubMed Central PMCID: PMCPMC8117967.

12. Chemaitelly H, Yassine HM, Benslimane FM, Al Khatib HA, Tang P, Hasan MR, et al. mRNA-1273 COVID-19 vaccine effectiveness against the B.1.1.7 and B.1.351 variants and severe COVID-19 disease in Qatar. Nature medicine. 2021;27(9):1614-21. Epub 2021/07/11. doi: 10.1038/s41591-021-01446-y. PubMed PMID: 34244681.

13. National Project of Surveillance for Variants of Concern and Viral Genome Sequencing. Qatar viral genome sequencing data. Data on randomly collected samples. <https://www.gisaid.org/phylodynamics/global/nextstrain/> 2021. Available from: <https://www.gisaid.org/phylodynamics/global/nextstrain/>.

14. Benslimane FM, Al Khatib HA, Al-Jamal O, Albatesh D, Boughattas S, Ahmed AA, et al. One Year of SARS-CoV-2: Genomic Characterization of COVID-19 Outbreak in Qatar. Front Cell Infect Microbiol. 2021;11:768883. Epub 2021/12/07. doi: 10.3389/fcimb.2021.768883. PubMed PMID: 34869069; PubMed Central PMCID: PMCPMC8637114.

15. Hasan MR, Kalikiri MKR, Mirza F, Sundararaju S, Sharma A, Xaba T, et al. Real-Time SARS-CoV-2 Genotyping by High-Throughput Multiplex PCR Reveals the Epidemiology of the Variants of Concern in Qatar. Int J Infect Dis. 2021;112:52-4. Epub 2021/09/16. doi: 10.1016/j.ijid.2021.09.006. PubMed PMID: 34525398.

16. Saththasivam J, El-Malah SS, Gomez TA, Jabbar KA, Remanan R, Krishnankutty AK, et al. COVID-19 (SARS-CoV-2) outbreak monitoring using wastewater-based epidemiology in Qatar. Sci Total Environ. 2021;774:145608. Epub 2021/02/20. doi: 10.1016/j.scitotenv.2021.145608. PubMed PMID: 33607430; PubMed Central PMCID: PMCPMC7870436.

17. El-Malah SS, Saththasivam J, Jabbar KA, K KA, Gomez TA, Ahmed AA, et al. Application of human RNase P normalization for the realistic estimation of SARS-CoV-2 viral load in wastewater: A perspective from Qatar wastewater surveillance. Environ Technol Innov. 2022;27:102775. Epub 2022/06/29. doi: 10.1016/j.eti.2022.102775. PubMed PMID: 35761926; PubMed Central PMCID: PMCPMC9220754.

18. Tang P, Hasan MR, Chemaitelly H, Yassine HM, Benslimane FM, Al Khatib HA, et al. BNT162b2 and mRNA-1273 COVID-19 vaccine effectiveness against the SARS-CoV-2 Delta variant in Qatar. Nature medicine. 2021;27(12):2136-43. Epub 2021/11/04. doi: 10.1038/s41591-021-01583-4. PubMed PMID: 34728831.

19. Chemaitelly H, Ayoub HH, AlMukdad S, Coyle P, Tang P, Yassine HM, et al. Duration of mRNA vaccine protection against SARS-CoV-2 Omicron BA.1 and BA.2 subvariants in Qatar. Nature communications. 2022;13(1):3082. Epub 2022/06/03. doi: 10.1038/s41467-022-30895-3. PubMed PMID: 35654888.

20. Qassim SH, Chemaitelly H, Ayoub HH, AlMukdad S, Tang P, Hasan MR, et al. Effects of BA.1/BA.2 subvariant, vaccination and prior infection on infectiousness of SARS-CoV-2 omicron infections. J Travel Med. 2022;29(6). Epub 2022/06/01. doi: 10.1093/jtm/taac068. PubMed PMID: 35639932; PubMed Central PMCID: PMCPMC9213851.

21. Altarawneh HN, Chemaitelly H, Ayoub HH, Hasan MR, Coyle P, Yassine HM, et al. Protective effect of previous SARS-CoV-2 infection against Omicron BA.4 and BA.5 subvariants. The New England journal of medicine. 2022;387(17):1620-2. Epub 2022/10/06. doi: 10.1056/NEJMc2209306. PubMed PMID: 36198139; PubMed Central PMCID: PMCPMC9559315.

22. Chemaitelly H, Tang P, Coyle P, Yassine HM, Al-Khatib HA, Smatti MK, et al. Protection against Reinfection with the Omicron BA.2.75 Subvariant. N Engl J Med. 2023;388(7):665-7. Epub 2023/01/19. doi: 10.1056/NEJMc2214114. PubMed PMID: 36652342; PubMed Central PMCID: PMCPMC9878583.

23. World Health Organization (WHO). Living guidance for clinical management of COVID-19. Aavailable from: <https://www.who.int/publications/i/item/WHO-2019-nCoV-clinical-2021-2>. Accessed on: February 27, 2023. 2023.

24. World Health Organization (WHO). International Guidelines for Certification and Classification (Coding) of COVID-19 as Cause of Death. Available from: <https://www.who.int/publications/m/item/international-guidelines-for-certification-and-classification-(coding)-of-covid-19-as-cause-of-death>. Accessed on: February 27, 2023. 2023.

25. Li R, Liu H, Fairley CK, Ong JJ, Guo Y, Lu P, et al. mRNA-based COVID-19 booster vaccination is highly effective and cost-effective in Australia. Vaccine. 2023;41(15):2439-46. Epub 2023/02/14. doi: 10.1016/j.vaccine.2023.01.075. PubMed PMID: 36781332; PubMed Central PMCID: PMCPMC9894775.

26. Li R, Liu H, Fairley CK, Zou Z, Xie L, Li X, et al. Cost-effectiveness analysis of BNT162b2 COVID-19 booster vaccination in the United States. Int J Infect Dis. 2022;119:87-94. Epub 2022/03/27. doi: 10.1016/j.ijid.2022.03.029. PubMed PMID: 35338008; PubMed Central PMCID: PMCPMC8938315.

27. Baggett TP, Scott JA, Le MH, Shebl FM, Panella C, Losina E, et al. Clinical Outcomes, Costs, and Cost-effectiveness of Strategies for Adults Experiencing Sheltered Homelessness During the COVID-19 Pandemic. JAMA Network Open. 2020;3(12):e2028195-e. doi: 10.1001/jamanetworkopen.2020.28195.

28. Neilan AM, Losina E, Bangs AC, Flanagan C, Panella C, Eskibozkurt GE, et al. Clinical Impact, Costs, and Cost-effectiveness of Expanded Severe Acute Respiratory Syndrome Coronavirus 2 Testing in Massachusetts. Clin Infect Dis. 2021;73(9):e2908-e17. Epub 2020/09/19. doi: 10.1093/cid/ciaa1418. PubMed PMID: 32945845; PubMed Central PMCID: PMCPMC7543346.

29. Reddy KP, Shebl FM, Foote JHA, Harling G, Scott JA, Panella C, et al. Cost-effectiveness of public health strategies for COVID-19 epidemic control in South Africa: a microsimulation modelling study. Lancet Glob Health. 2021;9(2):e120-e9. Epub 2020/11/15. doi: 10.1016/S2214-109X(20)30452-6. PubMed PMID: 33188729; PubMed Central PMCID: PMCPMC7834260.

30. Global Burden of Disease Collaborators. Global, regional, and national incidence, prevalence, and years lived with disability for 354 diseases and injuries for 195 countries and territories, 1990-2017: a systematic analysis for the Global Burden of Disease Study 2017. Lancet. 2018;392(10159):1789-858. Epub 2018/11/30. doi: 10.1016/S0140-6736(18)32279-7. PubMed PMID: 30496104; PubMed Central PMCID: PMCPMC6227754.

31. Cleary SM, Wilkinson T, Tamandjou Tchuem CR, Docrat S, Solanki GC. Cost-effectiveness of intensive care for hospitalized COVID-19 patients: experience from South Africa. BMC Health Serv Res. 2021;21(1):82. Epub 2021/01/24. doi: 10.1186/s12913-021-06081-4. PubMed PMID: 33482807; PubMed Central PMCID: PMCPMC7820836.

32. Kohli M, Maschio M, Becker D, Weinstein MC. The potential public health and economic value of a hypothetical COVID-19 vaccine in the United States: Use of cost-effectiveness modeling to inform vaccination prioritization. Vaccine. 2021;39(7):1157-64. doi: <https://doi.org/10.1016/j.vaccine.2020.12.078>.

33. Sassi F. Calculating QALYs, comparing QALY and DALY calculations. Health Policy Plan. 2006;21(5):402-8. Epub 2006/08/01. doi: 10.1093/heapol/czl018. PubMed PMID: 16877455.

34. CCEMG and EPPI. The Campbell and Cochrane Economics Methods Group (CCEMG) and the Evidence for Policy and Practice Information Center Cost conversion tool. Available from: <https://eppi.ioe.ac.uk/costconversion/>. Accessed on: October 1, 2023. 2023.

35. Briggs AH, Goldstein DA, Kirwin E, Meacock R, Pandya A, Vanness DJ, et al. Estimating (quality-adjusted) life-year losses associated with deaths: With application to COVID-19. Health Econ. 2021;30(3):699-707. Epub 2020/12/29. doi: 10.1002/hec.4208. PubMed PMID: 33368853.
